# A health and economic evaluation of the spatial spillover effect from measles resurgence

**DOI:** 10.1038/s41598-025-21097-0

**Published:** 2025-10-14

**Authors:** Kexin Xie, Achla Marathe, Mugdha Thakur, Jiangzhuo Chen, Xinwei Deng, Anil Vullikanti

**Affiliations:** 1 Department of Statistics, Virginia Tech, Blacksburg, VA USA; 2 Biocomplexity Institute, University of Virginia, Charlottesville, VA 22903 USA; 3 Department of Public Health Sciences, University of Virginia, Charlottesville, VA USA; 4 Department of Computer Science, University of Virginia, Charlottesville, VA USA

**Keywords:** MMR vaccination rates, Spatial autoregressive models, Spatial spillover effects, Measles burden, Public health policy, Health care economics, Public health, Epidemiology, Health policy, Health care, Infectious diseases, Viral infection

## Abstract

The administration of the Measles, Mumps, and Rubella (MMR) vaccination has had a substantial impact on controlling the spread of measles on a global scale. Nevertheless, the COVID-19 pandemic caused major disruptions to normal immunization schedules, causing the omission or delay of routine immunizations. Expanding on previous research that simulated measles outbreaks using a detailed agent-based model, this study integrates epidemiological forecasts with spatial econometrics analysis. Our objective is to quantify the household-level direct and indirect health and economic impact of measles outbreaks caused by reduction in MMR vaccine uptake. A network-based SEIR (susceptible-exposed-infected-recovered) model is used to simulate the transmission of measles over a synthetic social contact network of Virginia, under various scenarios. Household-level costs of measles outbreak, encompassing MMR vaccine expenses, treatment costs, and productivity losses, are estimated from the simulation results. A Generalized Spatial Autoregressive (GSAR) model is used to estimate the spatial ‘spillover effect’ on neighboring counties. Our findings indicate that reduced MMR vaccination rates are associated with increased measles cases and related economic costs, which are intensified by disease transmissibility and moderated by home quarantine. The GSAR model, with spatial lag coefficients, shows significant spatial interdependencies. A small decrease in vaccination rate in an urban region like Richmond, Virginia, has significant economic and epidemiological spillover effect, while similar reductions in rural regions like Highland County, Virginia, have a negligible impact. A decline in MMR vaccination rate has ramifications for both disease incidence and the economy, presenting diverse consequences influenced by regional disparities. Policymakers should acknowledge the interconnectedness of health and economic outcomes across regions. This research underscores the necessity of implementing broad, region-wide policy measures in response to fluctuations in vaccination rates, prioritizing overarching strategies over localized interventions.

**Supplementary Information:**

The online version contains supplementary material available at 10.1038/s41598-025-21097-0.

## Introduction

The introduction of the Measles, Mumps, and Rubella (MMR) vaccine marked a pivotal milestone in public health, substantially reducing the global burden of these diseases. In 2000, the US officially declared measles eliminated, defined by an absence of sustained disease transmission for over a year^1^. However, the COVID-19 pandemic and subsequent lockdown measures posed challenges to public health, affecting routine childhood immunization programs like the MMR vaccine. According to a recent report from the World Health Organization and the Centers for Disease Control and Prevention, global coverage estimates for the first measles-containing vaccine dose increased from 72% to 86% during 2000–2019^2^. However, during the COVID-19 pandemic, it declined to 81% in 2021 across all regions. In 2022, global coverage improved to 83%, which still remained below 2019 levels^2^. Declining MMR vaccination rates nearly cost the United States its measles-free status. By end of 2024, there had already been 284 measles cases and 16 outbreaks (defined as 3 or more related cases), whereas in all of 2023 there were a total of 58 cases, indicating a concerning trend in the resurgence of measles in the US^3^.

The resurgence of measles is influenced by a range of complex, dynamic factors. Not only have widespread disruptions like COVID-19 broadly affected vaccination rates, but significant regional differences in coverage also remain—a phenomenon known as spatial heterogeneity. This uneven distribution creates geographic pockets of vulnerability, as areas with lower vaccination rates are more susceptible to outbreaks, undermining national efforts to eliminate the disease. Moreover, beyond vaccination coverage, additional factors such as demographic structures, mobility patterns, and varying levels of compliance with public health measures play crucial roles in shaping measles transmission^4,5^.

Considering the above facts, Thakur et al.^6^ conducted a study using a highly resolved agent-based model (ABM) to simulate measles outbreaks in Virginia during the pre-COVID and post-lockdown separately. Their simulations encompassed a range of scenarios, from varied transmission intensities to differential adherence to stay-home interventions, spatially distinct immunization rates, and varying degrees of MMR vaccination rate reductions. Their findings underscored the implications of diminishing immunization rates on measles incidence, and scenarios where even quick isolation of cases and higher compliance with home-isolation interventions were not enough to control the outbreak^6^.

Historically, measles outbreaks have posed complicated challenges. They have not only affected public health systems but also exerted extensive economic strain. Hospitalizations, medical treatments, public health responses, and the indirect costs associated with lost productivity and school closures contribute to the broader economic burden of measles outbreaks^7^. While Thakur et al.^6^ effectively detailed health impacts, their study did not analyze the associated economic burden. Additionally, the high computational demands of ABM simulations can limit the feasibility of examining a broad range of scopes or parameter configurations within a single study.

Building on the simulation of Thakur et al.^6^, our study employs a spatial economic metamodel as a complement to the ABM findings^8,9^. This approach enables us to explore associations between model parameters and spatial economic outcomes. This work evaluates the health and household-level economic effects (*direct* and *spillover*) of the potential resurgence of measles due to decreasing MMR vaccination rates. In this context, the *direct effect* is defined as the impact of changing vaccination coverage within a region on the infection rate in that same region. In contrast, the *spillover or the indirect effect* is defined as the impact of changes in vaccination coverage in one region on the infection rates in geographically neighboring regions^10–12^. While prior studies have investigated the direct effect of vaccination coverage variability^13–15^, the literature has offered limited insights into the spillover effect due to such variability. To quantify these effects and bridge the literature gap, our study provides a comprehensive evaluation of the regional economic implications of changes in MMR vaccination rates across specific geographic areas. Using the state of Virginia as a case study, we applied a spatial econometric model to incorporate commuter flow data for estimating spillover effects caused by a decrease in vaccination rate in one county to other counties.

Traditional spatial econometric models, however, are limited by the normality assumption in response variables. Lambert et al.^16^ extended the traditional models through the development of a Poisson Spatial Autoregressive Model for count data using a two-step limited information maximum likelihood approach. LeSage et al.^17^ developed a Spatial Probit model implemented by a Bayesian Markov chain Monte Carlo estimation. Subsequently, Bivand et al.^18,19^ introduced a spatial lag autoregressive component in Integrated Nested Laplace Approximations (INLA) under a Bayesian framework, enhancing the model’s flexibility and applicability to various data structures and distributions. Leveraging INLA, we propose the Generalized Spatial Autoregressive Model (GSAR) with a Gamma distribution for cost data and a Poisson distribution for measles-count data, offering a more suitable approach for non-Gaussian data.

By bridging epidemiological projections with spatial analysis, our aim is to provide evidence-based insights that can guide policymaking. Our contributions are threefold: (1) we provide an assessment of the economic consequences of measles outbreaks, incorporating direct and indirect costs; (2) we quantify spatial spillover effects using commuter flow data, highlighting regional interdependencies; and (3) we propose a flexible GSAR framework for non-Gaussian data, enhancing the methodological toolkit for spatial econometric analysis. Using Virginia as a case study, our findings offer valuable insights for policymakers to mitigate the consequences of declining vaccination rates through regionally coordinated strategies.

## Methodology

### Network-based SEIR model and data collection

**Fig. 1:**
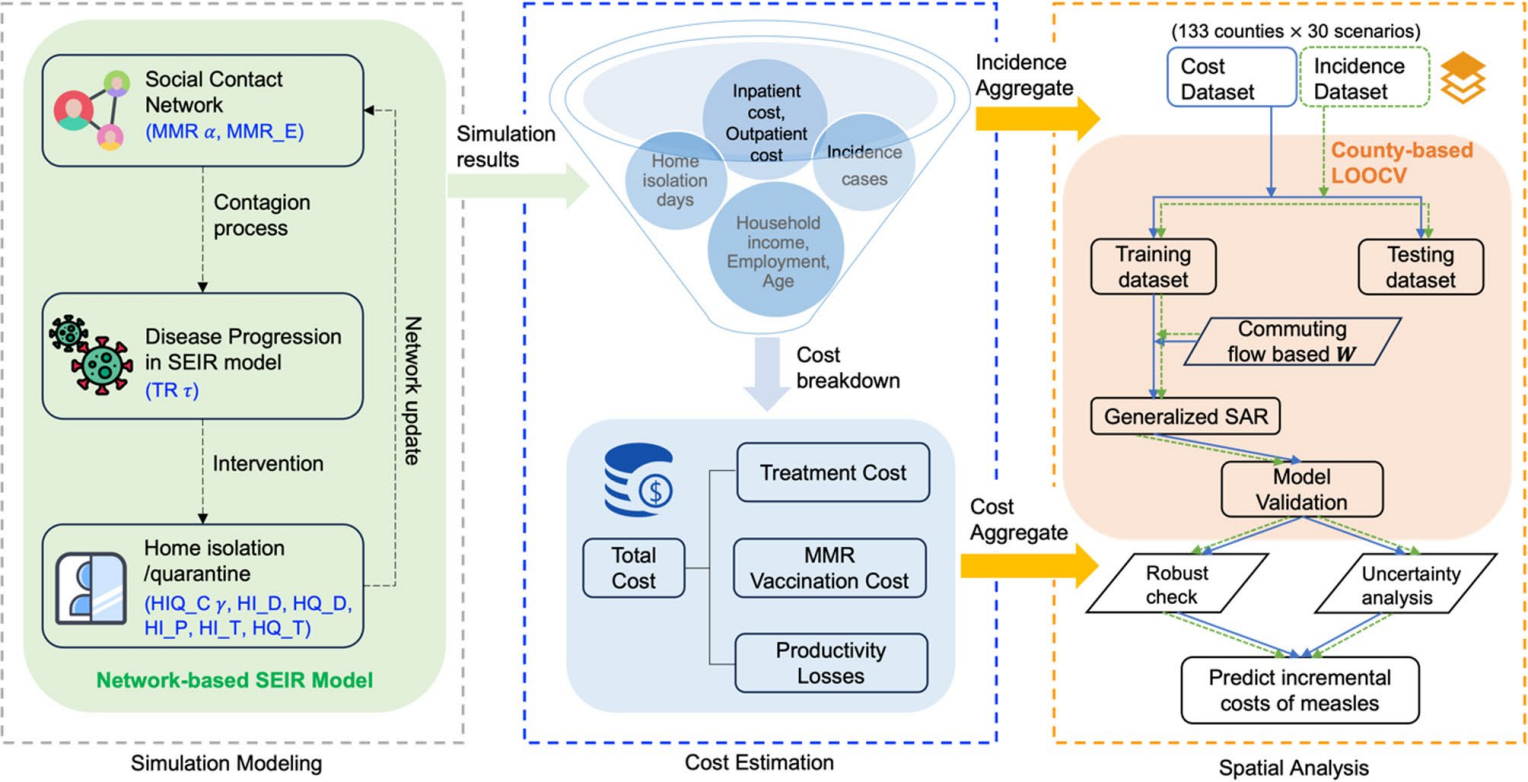
Flowchart of the outline of data analysis methodology. HIQ_C$\gamma$, Home isolation/quarantine compliance; HI_D, Home isolation delay; HQ_D, Home quarantine of delay; HI_P, Home isolation adherence probability; MMR_E, Effectiveness of MMR vaccine; HI_T, Duration of home isolation; HQ_T, Duration of home quarantine; TR$\tau$, Transmissibility rate; MMR$\alpha$, MMR level reduction.

Our study builds on the findings of Thakur et al.^6^. The authors modeled potential trajectories of measles resurgence in Virginia for a relatively short time duration (365 days) under two distinct scenarios: (1) pre-COVID-19 and (2) post-lockdown conditions where social activities return to pre-COVID-19 levels, but MMR immunization rates decline. In this study, we use the same scenarios but focus on estimating the economic burden of resurgence in each of the scenarios and the spillover effects. The outline of the analyses is illustrated through flowchart in Fig. 1.

An agent-based model simulates the spread of measles in Virginia (VA). The disease spreads across a synthetic social contact network of VA, where nodes represent individuals and edges represent contacts between individuals. There are approximately 7.6 million nodes and 371.9 million edges in the network. These individuals have demographic attributes as available in the US Census and are placed in households that are geo-located^20–22^. Their daily activities are determined from time-use surveys and geolocation sources, forming the basis for person-to-person contacts and disease transmission. Using this network, the model simulated the transmission of measles with an SEIR (Susceptible-Exposed-Infected-Recovered) model with several critical parameters influencing the outcomes. Each simulation begins with one random infected node of age 5–17 years and is run for 365 time-steps (365 days). For more details on this agent-based model and the construction of the synthetic contact network, see Thakur et al.^6,20–22^.

For our study, simulated outcomes on measles infection counts and the number of days individuals spend in home isolation or quarantine are derived from the above agent-based model. There are a total of 30 distinct scenarios, as outlined in Table 1. For each scenario, 300 replicates are generated, resulting in a total of 9000 simulations. The disease starts from a single index case that is randomly selected in each replicate. The economic evaluation is based on the integration of disease outcomes, cost estimates, along with the demographic characteristics of each household.

**Table 1:** Parameters and their descriptions for the 30 scenarios: 3 scenarios are Pre-covid, 27 scenarios are Post-covid^6^.

Parameter	Pre-covid experiments values	Post-covid experiments values
Transmissibility ($\tau$)	0.5^1^	{0.4, 0.5, 0.6}
MMR reduction rate ($\alpha\:\%$)	Base case 0%^2^	{5, 15, 25}%^3^
Parameter	Consistent experiments values in pre-covid and post-covid	
Home isolation/quarantine compliance ($\gamma\:\%$)	{75, 85, 95}%	
Home isolation delay	3 days	
Home quarantine of delay	3 days (household members) 1 day after being traced (contacts)	
Effectiveness of MMR vaccine	100% (assumed)	
Duration of home isolation	7 days (until recovered)	
Simulation duration	365 days (assumed)	

^1^Calibrated to generate an outbreak size of 649, which scales the number of cases to the population size in Virginia, to match the 605 reported measles cases during the 2018–2019 outbreak in New York City^23^. The NYC outbreak is chosen as a relevant benchmark because it was the most recent and only large-scale outbreak in the U.S. with a population size comparable to that of Virginia. For details of the calibration procedure, see Thakur et al. (2022)^6^.

^2^The base-case scenario assumes the overall vaccination rate of the Virginia population to be 91.5% for both adults and children^6^.

^3^The decline in the MMR rate in the population is assumed to be uniformly distributed across Virginia.

### Cost estimation

We assess the economic impact of measles across 133 counties in Virginia, which include 38 independent cities, from a societal perspective. For each county, we evaluate the cost of measles outbreak for each of the 9000 simulation runs. Following Pike et al.^24^, costs are valued on an incremental resource-use basis and comprise treatment (direct medical) treatment, productivity losses, and MMR vaccination cost. Our objective is to quantify the direct societal burden attributable to cases and vaccination. Further assumptions and parameterization are detailed in subsequent sections. All costs are stated in 2021 US dollars (USD). All dollar amounts were modeled in 2021 USD to preserve internal consistency with simulation inputs; readers can convert any reported total to later-year USD. This currency rescaling does not affect comparative or spatial conclusions.

#### Treatment costs

The treatment costs are a function of the incidence of measles. Based on the historical data, the US Center for Disease Control and Prevention (CDC) has estimated that approximately 1 in 4 cases in the US culminate in hospitalization, with 1 in 1000 escalating to fatalities^25^. For the purpose of computation, the treatment cost per case, denoted as ${C}^{m}$, is calculated using the formula:

1 $$\:\begin{array}{c}{C}^{m}=\frac{1}{4}\times\:{C}^{hosp}+\:\frac{3}{4}\times\:{C}^{outpatient},\end{array}$$

where the cost of hospitalization is assumed to vary between $4,032 to $46,060 per case, and the cost of outpatient visit varies from $88 to $526 per case, based on data (in 2013 US$) from Whitney et al.^26,27^. These unit-cost values are derived from U.S. insurance claims databases and reflect realized reimbursements from payers to providers for measles-related care. We use them as a proxy for direct medical costs from a societal perspective, that is, medical services and supplies associated with outpatient visits and hospitalizations, including laboratory services and prescription drugs. Non-medical costs such as patient travel/time, money spent on food and lodging etc. are not consistently captured in claims and are therefore excluded. Our focus remains on estimating the spatial spillover effects. For our computations, we use the median of the reported ranges for our baseline estimates and inflated to 2021 US$: $${C}^{hosp}=\$\text{29,132.92}$$ and $${C}^{outpatient}=\$357.10$$. The median offers a more robust central estimate under such skewed distributions and ensures that the results are not disproportionately influenced by extreme values.

#### Productivity losses

Productivity loss is estimated to occur when working adults must stay home from work either to take care of themselves or a child, due to sickness. If an individual is working, the wage loss is equal to daily wages multiplied by the number of missed working days. To calculate daily wages, we use household’s annual income and divide it by the number of working days in a year (i.e. 261), and then divide it by the number of working individuals in the household.

Productivity loss due to a child staying home is estimated in the following manner. If a non-working individual aged 13 or above is available within the household, that individual is designated as the caregiver for the child, and there is no loss in wages. In the absence of a non-working family member, the role of a caregiver is assigned to a working family member, and the wage loss for this member is calculated the same way as before.

To methodically quantify the daily productivity loss for each working individual $i$ within household $j$, denoted as (${C}_{i,j}^{p}$), we employ the following formulation:

2 $$\:\begin{array}{c}{C}_{i,j}^{p}=\frac{\text{H}\text{o}\text{u}\text{s}\text{e}\text{h}\text{o}\text{l}\text{d}\:\text{i}\text{n}\text{c}\text{o}\text{m}\text{e}}{\#\text{W}\text{o}\text{r}\text{k}\text{e}\text{r}\text{s}\:\text{i}\text{n}\:\text{h}\text{o}\text{u}\text{s}\text{e}\text{h}\text{o}\text{l}\text{d}}\times\:\frac{1}{261}, \end{array}$$

where the denominator 261 represents the average number of working days in a year. In the absence of data on individual income, we use households’ annual income and the number of employed family members to determine the productivity loss.

#### MMR vaccination cost

We assume MMR doses are co-administered during routine well-child visits, so no incremental administration, travel, or additional-visit costs are attributed in the base case. We use a transparent retail price per MMR dose because CDC VFC contract prices apply to awardee programs and are not directly available to private providers. Since programs like VFC can procure at discounted contract rates, our retail assumption is conservative. Drawing from the data provided by GoodRx^28^, each dose of the MMR vaccine is priced at $114. Given the CDC’s recommendation for two doses per individual—with the initial dose administered between 12 and 15 months and the subsequent dose between 4 and 6 years^29^—the total cost for the full vaccination schedule per individual amounts to $228, i.e., $${C}^{v}=\$228$$. Occasional stand-alone vaccination visits that could generate extra administration or non-medical costs are assumed rare and negligible in this setting and are excluded.

#### Total cost

We assume that every measles infection is reported and treated as either an inpatient or outpatient. Using the above parameters, the total cost for county $s$, denoted by ${T}_{s}$, is calculated as:

3 $$\:\begin{array}{c}\:{T}_{s}={C}^{m}\times\:{I}_{s}+{C}^{v}\times\:\:{V}_{s}+\:\sum\:_{j=1}^{{N}_{s}}\sum\:_{i=1}^{{n}_{j}}{C}_{i,j}^{p}\times\:{t}_{i,j},\end{array}$$

where ${I}_{s}$ is the number of infected measles case in county $\:s$; $\:{V}_{s}$ is the number of vaccinated individuals in county $s$; ${t}_{i,j}$ is the number of days of home stay of working individual $i$ in household $j$; ${n}_{j}$ is the total number of working individuals in household $j$; ${N}_{s}$ is the total number of households in county $s$.

### Spatial analysis

We use a spatial econometric model to examine the epidemiological and economic cost of measles outbreak in the county of origin as well as its impact in the neighboring regions due to spatial interdependencies between counties. We measure (A) the mean total measles cases to estimate the average epidemiological impact and (B) the mean total cost per capita to estimate the average financial burden. We also evaluate the 90th percentiles of measles cases and total cost per capita, to understand the variability in outcomes across runs. See details in the Supplementary Appendix S4, and results in the Table S6 and Figure S5–S6.

#### Generalized spatial autoregressive model (GSAR)

To understand the spatial complexities inherent in the spread of measles, it is imperative to acknowledge the spatial interdependence between regions. An alteration in one region can instigate changes in its neighboring regions, a cascading effect often referred to as the ‘spillover effect’. Addressing this requires a modeling approach that can capture such spatial dependence.

The response variable represents measles cases or standardized costs across counties $\mathcal{S}=(\text{1,2},\dots\:,s)$, denoted as $\varvec{y}=({y}_{1},\dots\:{y}_{s})$. The outcome ${y}_{i}$ is assumed to come from a distribution of the Exponential family with mean parameter ${\mu\:}_{i}$. The relationship between ${\mu\:}_{i}$ and the linear predictor on a vector of covariates ${\varvec{x}}_{\varvec{i}}$ is established through a link function $g(\cdot\:)$:

4 $$\:\begin{array}{c}g\left({\mu\:}_{i}\right)={\eta\:}_{i}={{\varvec{x}}_{\varvec{i}}}^{T}\beta\:. \end{array}$$

To examine the spatial relationships between regions, we use a spatial weight metric constructed from the commuter flows between counties in Virginia. We use county-to-county commuting flows from 2016-2020 5-Year ACS Census data^30^. The commute flow based spatial weight matrix, $\varvec{W}$, is formulated with each element, ${w}_{ij}$, defined as:

5 $$\:\begin{array}{c}{w}_{ij}=\left\{\begin{array}{c}\frac{{c}_{ij}}{\sqrt{{N}_{i}{N}_{j}}},\:\:i\ne\:j\\\:0,\:\:i=j,\end{array},\right.\: \end{array}$$

where ${c}_{ij}$ is the number of individuals commuting from one specific region $i$ to another region $j$; ${N}_{i}$ and ${N}_{j}$ indicate the population of region $i$ and $j$, respectively. Dividing ${c}_{ij}$ by the square root of ${N}_{i}{N}_{j}$ normalizes commuter flows relative to the populations of the regions, ensuring the weights represent proportional interactions rather than being skewed by absolute population sizes. Notably, $\varvec{W}\:$is standardized such that the sum of all its elements equals 1, maintaining uniformity in the weight matrix.

Under the Bayesian perspective, the Generalized Spatial Autoregressive Model (GSAR) is used to account for spatial dependence in data where outcomes in one region may be influenced by outcomes or predictors in neighboring regions. This is particularly relevant in infectious disease modeling, where disease spread is not confined to administrative boundaries. In the GSAR framework, the dependent variable $\varvec{y}$ is assumed to follow a distribution from the Exponential family. For example, if the dependent variable is assumed to follow a Poisson distribution, then

$$\:\varvec{y}\sim\text{P}\text{o}\text{i}\text{s}\left(\varvec{\mu\:}\right),\:$$

where $\varvec{\mu\:}$ is an $n\times\:1$ vector representing the expectation of $\varvec{y}$, i.e., $\varvec{\mu\:}=E\left(\varvec{y}\right)$. A link function $g\left(\varvec{\mu\:}\right)$ relates the expected outcome to a linear predictor $\varvec{\eta\:}$ via:

6 $$\:\begin{array}{c}g\left(\varvec{\mu\:}\right)=\varvec{\eta}\:={\left({\varvec{I}}_{\varvec{n}}-\rho\:\varvec{W}\right)}^{-1}\varvec{X}\varvec{\beta}\:, \end{array}$$

where $\varvec{X}$ denotes an $n\times\:p$ matrix of independent variables, while $\varvec{\beta\:}$ signifies the $p\times\:1$ coefficient vector of the independent variables to be estimated. The matrix $\varvec{W}$ indicates the $n\times\:n$ spatial weight structure. The spatial lag coefficient, represented by $\rho$, is to be estimated and characterizes the spatial autocorrelation coefficient. The term ${\left({\varvec{I}}_{\varvec{n}}-\rho\:\varvec{W}\right)}^{-1}$introduces spatial spillover effects, meaning that a change in a covariate in one region can influence outcomes in neighboring regions through the spatial structure defined by the weight matrix $\varvec{W}$.

To derive approximate inference for this model, Goméz-Rubio et al.^31^ developed a latent class “slm” in **“INLA”** R-package^32^. For an in-depth exploration of the estimation process and its mathematical foundations, see Goméz-Rubio et al. (2021)^31^.

In our study, we employ the GSAR to independently analyze the incidences of measles cases and the associated measles costs. Specifically, the measles cases are modeled using a Poisson distribution with a logarithmic link function (GSAR-Poisson), reflecting their discrete nature and right-skewed characteristics. Concurrently, the cost data are modeled using a Gamma distribution, also integrated with a logarithmic link function (GSAR-Gamma), to accommodate their non-negative and skewed distributional properties.

#### Direct effect and spillover effect

It is crucial to distinguish the definitions of direct and spillover (indirect) effects within the Spatial Autoregressive (SAR) model from the conventional usage of these terms in the vaccine literature^33^. In the SAR model, these terms are defined differently due to the spatial interdependencies inherent in the model. Specifically, the SAR framework typically identifies two types of marginal effects: direct effects and spillover (or indirect) effects. The direct effect refers to the change in the dependent variable in a particular region resulting from a unit change in the explanatory variable within that same region. Conversely, the spillover effect, often termed the indirect effect, reflects the alteration in the dependent variable in one region due to a unit change in the explanatory variable in a neighboring region^34^.

The full detailed derivations for the direct and spillover effects are extensively documented in the Supplementary Appendix S1.1.

#### Variable selection

In the GSAR model, three experimental setting parameters serve as the primary explanatory variables: MMR vaccination reduction rate, transmissibility, and compliance with home isolation/quarantine directives. The MMR vaccination reduction rate is conceptualized as a continuous variable, while transmissibility and adherence directives are delineated as factor variables: {0.4, 0.5, 0.6} for measles transmissibility levels (low, baseline, high) and {75%, 85%, 95%} for home isolation/quarantine compliance levels (low, median, high), respectively.

To account for the potential confounders affecting the epidemiological and the economic impact of measles, the model integrates four demographic attributes of each county as covariates. These include average annual household income, the proportion of males, the proportion of children below 5 years of age, and Proportion of employed population. The selection of these confounders is informed by a rigorous assessment of multicollinearity and individual variable significance.

### Statistical analysis

From the 9000 simulation runs, we obtain simulated data on measles incidence, isolation/quarantine days, MMR vaccine costs, medical treatment costs, and productivity losses. To carry out the spatial analysis, the simulated results are aggregated by 133 counties and 30 unique experimental scenarios. To ascertain the presence of spatial dependency, we compare the GSAR model against a non-spatial generalized linear model (GLM), with additional diagnostic results reported in Supplementary Appendix S2.2 (Tables S2 and S3). As suggested by Kelejian and Prucha (2001)^35^, Morans’ I (Moran’s Index) statistic is utilized as a measure of spatial dependence. This statistic allows for the quantification of the linear relationship between observations and their spatially lagged counterparts. The value of Moran’s I tends to fall within the range of [− 1, 1]. A positive value is indicative of a positive spatial correlation, while a negative value suggests a negative spatial correlation. On the other hand, values close to 0 are associated with a lack of spatial autocorrelation. The observed Moran’s index value in our model indicates a significant spatial correlation, indicating strong spatial interdependencies within the region. Certain model fit indices, including Deviance Information Criterion (DIC), and Watanabe-Akaike Information Criterion (WAIC) are used to evaluate the fitness of the model. DIC is a generalization of AIC, developed especially for Bayesian model comparison^36^, and WAIC can be seen as an improvement over the DIC^37^.

To assess the predictive accuracy of the model, the Leave-One-Out Cross-Validation (LOOCV) approach is implemented to ensure consistency in representation. Note that we have 133 counties, and each county has 30 experimental scenarios, resulting in a total of 3990 observations. In this approach, each of the 133 counties is sequentially left out once for testing, while the model is trained on the remaining counties. This ensures that predictions are grounded on a comprehensive range of experimental settings from multiple counties. We compute the predictive Root Mean Square Error (PRMSE) around our forecasts. Furthermore, we also obtain the predictive intervals from the posterior predictive distribution which represents the probability of future observations given the observed data. These not only offer a clear measure of the prediction performance, but also provide insights into the range of possible outcomes.

Data analysis in this paper is performed using software R with Version 4.3.0. We use “spdep” package^38^ and “INLA” package^32^ to conduct the spatial correlation test and spatial econometric model, respectively.

## Results

### Disease outcomes and cost estimates

**Fig. 2:**
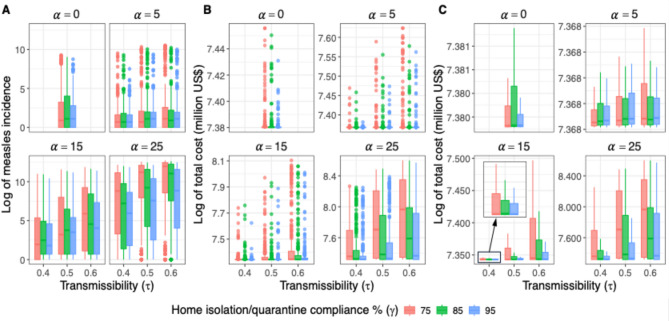
Effect on (**A**) measles incidence, (**B**) total costs (in million USD) with outliers, and (**C**) total costs (in million USD) without outliers in base-10 logarithmic scale of varying transmissibility $\:\left(\tau\right)$, home isolation compliance rate ($\gamma\:\%$), and the reduction in MMR vaccination rate ($\alpha\:\%$). The subplot headed $\alpha\:=0$ show the base case scenario when there is no reduction in vaccination rate and the transmission rate is set at 0.5.

The current analysis focuses on the following parameters: transmissibility ($$\tau$$), the decline in MMR vaccination rate ($$\alpha\:\%$$), and compliance with home isolation/quarantine rate ($$\gamma\:\%$$). Figure 2 shows a base-10 logarithmic scaled boxplot of measles cases and total cost in million US$ for each of the 30 scenarios. Each scenario was simulated 300 times to account for any stochastic variation and the average results over the 300 replicates are reported for each scenario. This figure displays the rise in measles incidence and the consequent economic burden, with diminishing vaccination coverage, particularly when the drop in MMR rate exceeds 15%. At a 5% reduction in vaccination coverage, a higher transmission rate does not result in a noticeable increase in measles cases, nor does a higher compliance with home isolation consistently lead to a reduction in incidence. Both incidence and total cost remain relatively stable across varying levels of transmission and compliance, suggesting limited sensitivity to these factors when vaccine coverage is high. However, when alpha rises to higher levels, higher transmissibility leads to substantial increases in both incidence and cost, while greater compliance with home isolation results in more noticeable reductions in cases. When reduction in vaccination rate is 25%, and the transmission rate is 0.5 or higher, even a 95% compliance is insufficient to control the incidence.

**Fig. 3:**
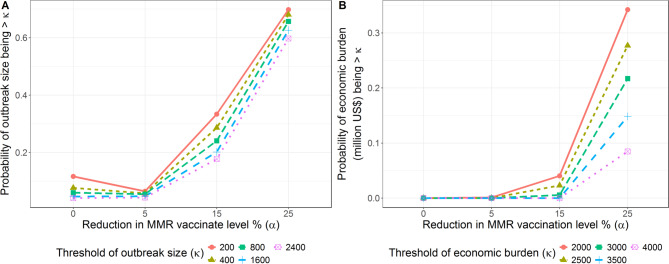
(**A**) The probability of outbreak size (**B**) the probability of economic burden (million USD) larger than a threshold κ (million USD).

However, numerous replicates with high measles rates and high economic burden are observed in Fig. 2, as explained by their high variance. Figure 3 shows how changes in $$\alpha$$ lead to changes in probabilities of having larger measles outbreaks and larger economic impact. Specifically, transitioning of reduction in vaccination rate from 0% to 25% results in a nearly sixfold increase in the likelihood of exceeding an outbreak size of 200. Similarly, on the economic front, the data reveal a stark increase in potential burden as $$\alpha$$ rises, evident at specific economic thresholds. For instance, at $$\alpha\:=0$$, the probability of surpassing economic threshold is negligible, even when the economic threshold is as high as 4000 million US$. But as reduction in vaccination rate increases to 25%, there is a significant increase in the likelihood of reaching or exceeding these thresholds.

**Fig. 4:**
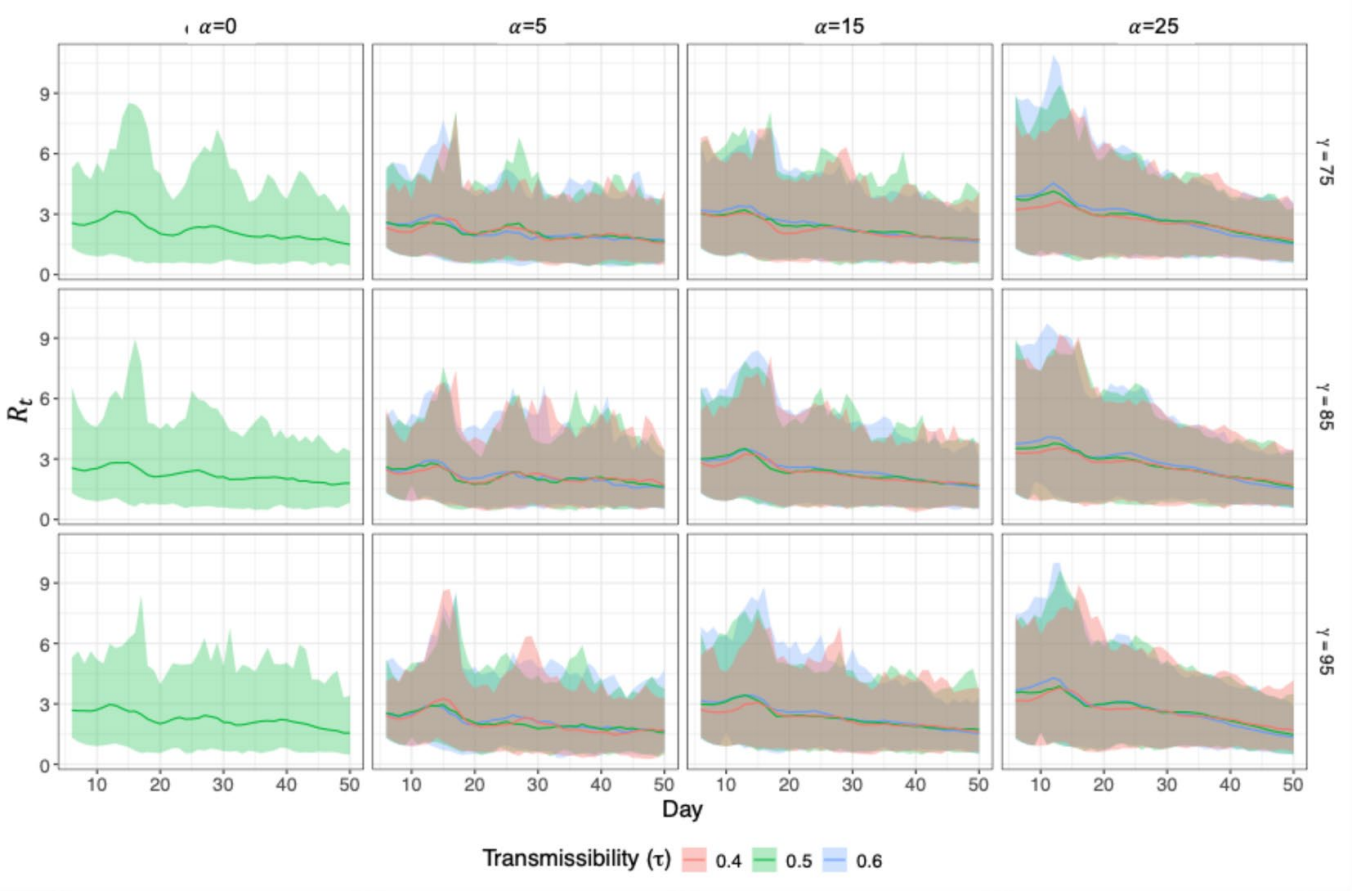
Temporal dynamics of the effective reproduction number (${R}_{t}$) of first 50 days of varying transmissibility $\left(\tau\:\right)$, home isolation compliance rate ($\gamma\:\%)$, and the reduction in MMR vaccination rate ($\alpha\:\%$). Solid lines represent the averaged ${R}_{t}$ over 300 replications; shaded ribbons indicate 95% confidence intervals. The subplot on the first column headed $\alpha\:=0$ show the base case scenario when there is no reduction in vaccination rate and the transmission rate is set at 0.5.

Figure 4 presents the daily effective reproduction number (${R}_{t}$) over the first 50 days of the outbreak under each of the 30 scenarios, aiming to directly reflect underlying transmission potential in the early stage of the outbreak. Each panel shows the averaged ${R}_{t}$ across 300 simulation replications as a solid line, with the shaded ribbon representing the 95% confidence interval. Estimates are generated using the “EpiEstim” R package (Cori et al.^39^), a well-established method that infers ${R}_{t}$ from observed incidence data combined with the serial interval distribution. In the baseline scenario ($\alpha\:=0$, $\tau\:=0.5$), where no vaccination coverage is lost, ${R}_{t}$ remains relatively low and stable across all transmissibility levels ($\tau$), consistently staying near or below 2. As vaccination coverage declines (increasing $\alpha$ from 5 to 25), we observe both an upward shift in the peak and greater variability, where ${R}_{t}$ frequently exceeds 4–5 early in the outbreak, suggesting accelerated epidemic growth. Interestingly, the effects of different transmissibility levels ($\tau$) and home isolation compliance ($\gamma$) appear comparatively negligible.

**Fig. 5:**
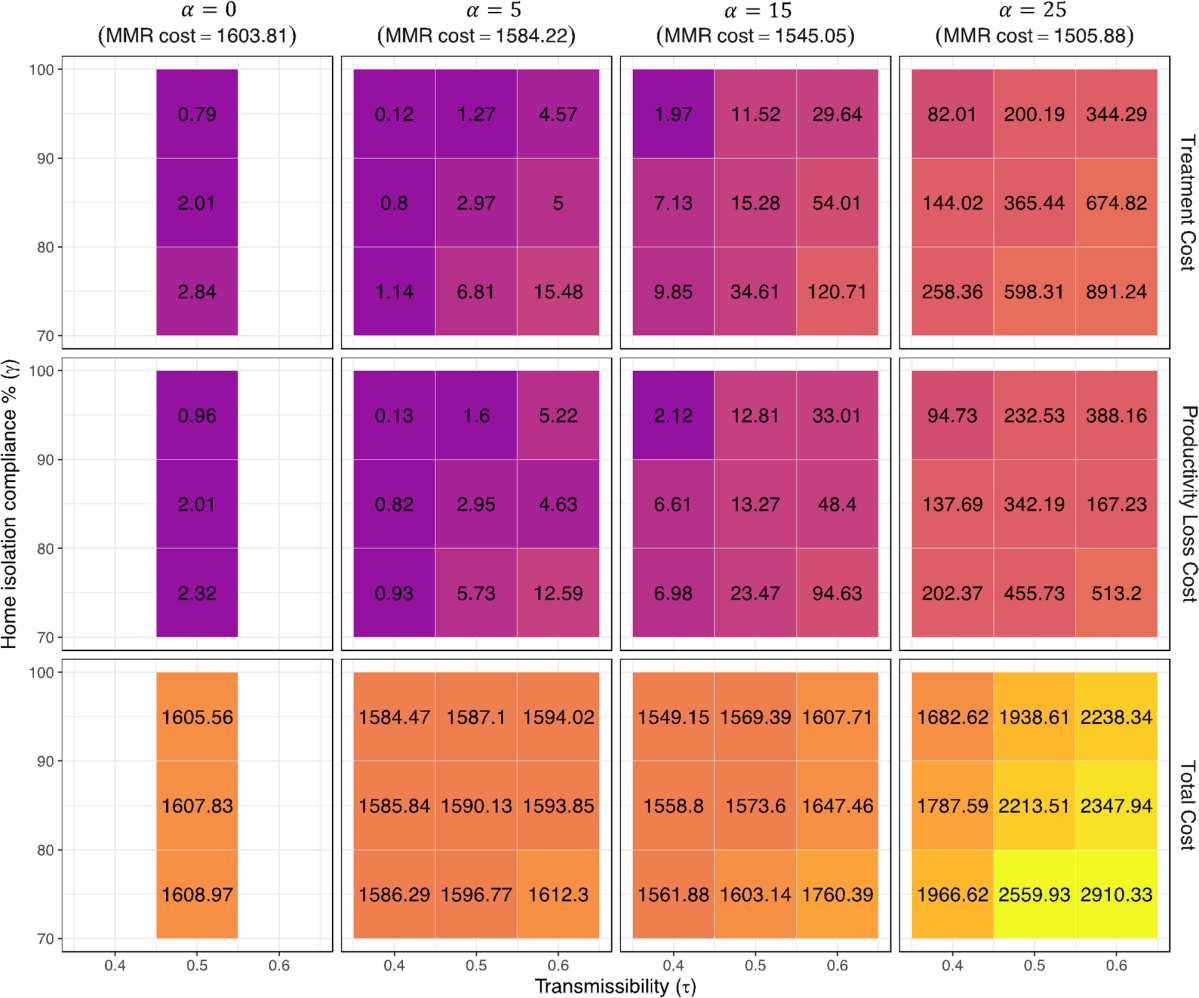
Mean of the treatment cost, productivity loss and total cost (in million US$) under various scenarios.

Figure 5 provides cost patterns for different categories in detail. Decreasing levels of MMR vaccination result in a decrease in vaccination costs. However, the simultaneous increases in both treatment costs and productivity loss offset these savings, resulting in an overall increase in costs, particularly when vaccination rates are below 85%. The present analysis clarifies that, when examining the levels of MMR vaccination in a particular population, there is a statistically significant increase in both medical and productivity costs when transmission rates are high. The data presented in our study also highlight the crucial need of adhering to social distancing protocols. Like the pattern observed in measles infections, when vaccination rates are lower, the effectiveness of quarantine measures becomes more pronounced. To provide an example, a reduction of 25% in vaccination rate combined with an increase in adherence to quarantine measures from 75% to 95% has the capacity to significantly reduce direct healthcare expenses by around 50%. In the base-case scenario, the predominant component of total cost is attributed to the expenses associated with MMR vaccine, accounting for almost 99% of the total expenditure. However, this number decreases to approximately 50% when accounting for reduction in vaccination rate, an increase in disease transmissibility, and higher compliance with isolation or quarantine protocols. Corresponding mean and standard deviation tables are provided in Supplementary Appendix Table S1.

### Results for spatial analysis

#### Generalized Spatial autoregressive model

Table 2 illuminates the distinctions in model efficacy between Non-Spatial and Spatial approaches for both (A) measles cases using Poisson regression, and (B) measles costs per capita employing Gamma regression. The GSAR models outperform their non-spatial counterparts for both measures, as reflected by lower DIC and WAIC values, indicating an improved fit that considers spatial heterogeneity. This superior fit is further substantiated by significantly lower PRMSE values, underscoring the GSAR models’ enhanced precision in forecasting outcomes. Notably, the incorporation of the Lag coefficient ($\rho$) in the GSAR models corrects for spatial autocorrelation, a critical factor in accurately estimating the spread and economic impact of measles, as also reflected in the reduced Moran’s I statistic.

**Table 2:** The estimation results on (A) measles cases and (B) measles costs per capita using Non-SAR models and GSAR models. Numbers in bold reflects that the parameter Estimation is significant at the 95% confidence level.

Variables	(A) Measles cases				(B) Measles costs per capita			
	Poisson		GSAR-Poisson		Gamma		GSAR-Gamma	
	Mean (SD)	95% CI	Mean (SD)	95% CI	Mean (SD)	95% CI	Mean (SD)	95% CI
(Intercept)	**− 11.338** (0.010)	(− 11.358, − 11.318)	**− 3.983** (0.142)	(− 4.289, − 3.708)	**5.332** (0.005)	(5.322, 5.342)	**1.866** (0.000)	(1.854, 1.867)
MMR level reduction % ($$\alpha$$)	**0.243** (0.000)	(0.242, 0.243)	**0.090** (0.004)	(0.082, 0.097)	**0.006** (0.000)	(0.006, 0.007)	**0.001** (0.000)	(0.001, 0.001)
Transmissibility ($$\tau$$)								
0.4 (Low level)	**− 0.897** (0.004)	(− 0.905, − 0.889)	**− 0.382** (0.042)	(− 0.466, − 0.300)	**− 0.057** (0.005)	(− 0.067, − 0.048)	**− 0.012** (0.004)	(− 0.019, − 0.005)
0.6 (High level)	**0.546** (0.003)	(0.540, 0.552)	**0.281** (0.038)	(0.207, 0.354)	**0.026** (0.005)	(0.016, 0.035)	0.006 (0.003)	(0.000, 0.003)
Home isolation/quarantine compliance % ($$\gamma$$)								
85 (Median level)	**− 0.422** (0.003)	(− 0.428, − 0.416)	**− 0.195** (0.038)	(− 0.269, − 0.121)	**− 0.036** (0.005)	(− 0.045, − 0.027)	**− 0.008** (0.003)	(− 0.014, − 0.001)
95 (High level)	**− 1.053** (0.004)	(− 1.060, − 1.046)	**− 0.434** (0.041)	(− 0.516, − 0.353)	**− 0.056** (0.005)	(− 0.065, − 0.047)	**− 0.012** (0.004)	(− 0.019, − 0.005)
Average household annual income (in thousand US$)	**0.169** (0.002)	(0.165, 0.174)	**0.060** (0.027)	(0.008, 0.113)	**0.028** (0.003)	(0.021, 0.034)	**0.007** (0.002)	(0.002, 0.012)
Proportion of male	**0.296** (0.002)	(0.291, 0.300)	**0.203** (0.020)	(0.163, 0.243)	**0.009** (0.002)	(0.005, 0.013)	**0.005** (0.002)	(0.002, 0.008)
Proportion of children under 5 years old	**0.017** (0.002)	(0.013, 0.021)	**0.085** (0.026)	(0.035, 0.136)	**0.015** (0.003)	(0.009, 0.020)	**0.007** (0.002)	(0.003, 0.011)
Proportion of employed population	**− 0.349** (0.004)	(− 0.357, − 0.342)	**− 0.212** (0.035)	(− 0.281, − 0.143)	**− 0.018** (0.004)	(− 0.025, − 0.010)	− 0.006 (0.003)	(− 0.011, 0.000)
DIC	101,215		8377.86		37,534		11,807	
WAIC	3,274,540		8108.16		37,541		10,829	
Predictive RMSE	17.004		0.878		5.806		0.139	
Moran’s I statistics	**0.169**		− 0.235		**0.899**		− 0.215	
Lag coefficient ($$\rho$$)			**0.717** (0.010)	(0.696, 0.736)			**0.686** (0.009)	(0.667, 0.703)

The reference level of transmissibility ($\tau$) is 0.5.

The reference level of home isolation/quarantine compliance % ($\gamma$) is 75.

Table 3 represents the marginal direct effects and spillover effects from GSAR models on measles cases and per capita costs. The results show that a percentage reduction in MMR levels (α) correlates with an increase in both measles cases and costs, with direct impacts being smaller yet consistent within the 95% confidence intervals. Interestingly, the indirect effects are larger, yet they follow the same direction as the direct effects, reinforcing the overall positive relationship between MMR level reduction and increases in cases and costs. For transmissibility (τ), at low levels, with respect to the base level of 0.5 transmissibility, we observe substantial negative direct and indirect impacts on measles cases and costs; at high levels of transmissibility, the model shows a trend with positive impacts that suggest an increase in both cases and costs as transmissibility intensifies. Home isolation/quarantine compliance percentage ($\gamma$) presents negative direct and indirect impacts for both median and high levels of compliance, indicating that higher compliance not only reduces the immediate burden of measles but also confers broader benefits. Furthermore, other demographic covariates such as average household annual income, proportion of males, and proportion of children under 5 years old exhibit significant positive direct and indirect impacts, while the proportion of the employed population has notable negative direct and indirect influence.

**Table 3:** Marginal direct impacts, indirect (Spillover) impacts and total impacts for (A) measles cases derived from GSAR-Poisson Model, and (B) measles costs per capita derived from GSAR-Gamma Model. Numbers in bold reflects that the parameter Estimation is significant at the 95% confidence level.

	(A) Measles cases—GSAR-Poisson			(B) Measles costs per capita—GSAR-Gamma		
	Direct impact (95% CI) $$\:\times\:{10}^{-4}$$	Indirect impact (95% CI) $$\:\times\:{10}^{-4}$$	Total impact (95% CI) $$\:\times\:{10}^{-4}$$	Direct impact (95% CI)	Indirect impact (95% CI)	Total impact (95% CI)
MMR level reduction % ($$\alpha$$)	**1.58** **(1.46**,** 1.69)**	**3.10** **(2.92**,** 3.28)**	**4.67** **(4.45**,** 4.89)**	**0.27** **(0.20**,** 0.35)**	**0.47** **(0.34**,** 0.61)**	**0.75** **(0.54**,** 0.96)**
Transmissibility ($$\tau$$)						
0.4 (Low level)	**− 6.75** **(− 8.23**,** − 5.24)**	**− 13.25** **(− 16.02**,** − 10.51)**	**− 19.99** **(− 24.22**,** − 15.74)**	**− 3.14** **(− 4.88**,** − 1.41)**	**− 5.44** **(− 8.47**,** − 2.46)**	**− 8.58** **(− 13.34**,** − 3.82)**
0.6 (High level)	**4.94** **(3.64**,** 6.21)**	**9.69** **(7.20**,** 12.29)**	**14.63** **(10.85**,** 18.50)**	1.62 (− 0.22, 3.44)	2.80 (− 0.39, 5.91)	4.42 (− 0.61, 9.36)
Home isolation/quarantine compliance % ($$\gamma$$)						
85 (Median level)	**− 3.43** **(− 4.62**,** − 2.15)**	**− 6.73** **(− 9.17**,** -4.25)**	**− 10.16** **(− 13.80**,** − 6.38)**	**− 1.93** **(− 3.55**,** − 0.15)**	**− 3.34** **(− 6.08**,** − 0.25)**	**− 5.28** **(− 9.65**,** − 0.40)**
95 (High level)	**− 7.63** **(− 9.10**,** − 6.19)**	**− 15.00** **(− 17.80**,** − 12.34)**	**− 22.63** **(− 26.86**,** − 18.49)**	**− 3.09** **(− 4.77**,** − 1.38)**	**− 5.35** **(− 8.29**,** − 2.44)**	**− 8.43** **(− 13.19**,** − 3.81)**
Average household annual income (in thousand US$)	**1.08** **(0.07**,** 2.03)**	**2.12** **(0.14**,** 3.86)**	**3.20** **(0.21**,** 5.79)**	**1.72** **(0.53**,** 2.83)**	**2.98** **(0.92**,** 4.91)**	**4.70** **(1.46**,** 7.79)**
Proportion of male	**3.55** **(2.84**,** 4.22)**	**6.97** **(5.52**,** 8.45)**	**10.52** **(8.43**,** 12.60)**	**1.31** **(0.53**,** 2.01)**	**2.27** **(0.87**,** 3.56)**	**3.58** **(1.39**,** 5.06)**
Proportion of children under 5 years old	**1.49** **(0.65**,** 2.36)**	**2.93** **(1.25**,** 4.73)**	**4.42** **(1.90**,** 7.11)**	**1.83** **(0.81**,** 2.77)**	**3.17** **(1.40**,** 4.88)**	**5.00** **(2.21**,** 7.67)**
Proportion of employed population	**− 3.72** **(− 5.05**,** − 2.52)**	**− 7.32** **(− 9.73**,** − 4.99)**	**− 11.04** **(− 14.73**,** − 7.58)**	**− 1.46** **(− 2.82**,** − 0.07)**	**− 2.53** **(− 4.96**,** − 0.12)**	**− 4.00** **(− 7.71**,** − 0.20)**

### Prediction results

The application of the Generalized Spatial Autoregressive (GSAR) model provides crucial advantages in forecasting the health and economic impact associated with changes in MMR vaccination rates. The GSAR captures spatial interdependencies, allowing for a clear understanding of direct and indirect economic ramifications.

We focus on predicting the potential impact of a 1% decrease in MMR vaccination rate in two specific regions in Virginia i.e. Richmond City and Highland County, using the GSAR model. The selection of these areas offers contrasting urban and rural perspectives, respectively. Richmond City is a densely populated urban area whereas Highland County is one of the least populous counties in Virginia and is rural.

**Fig. 6:**
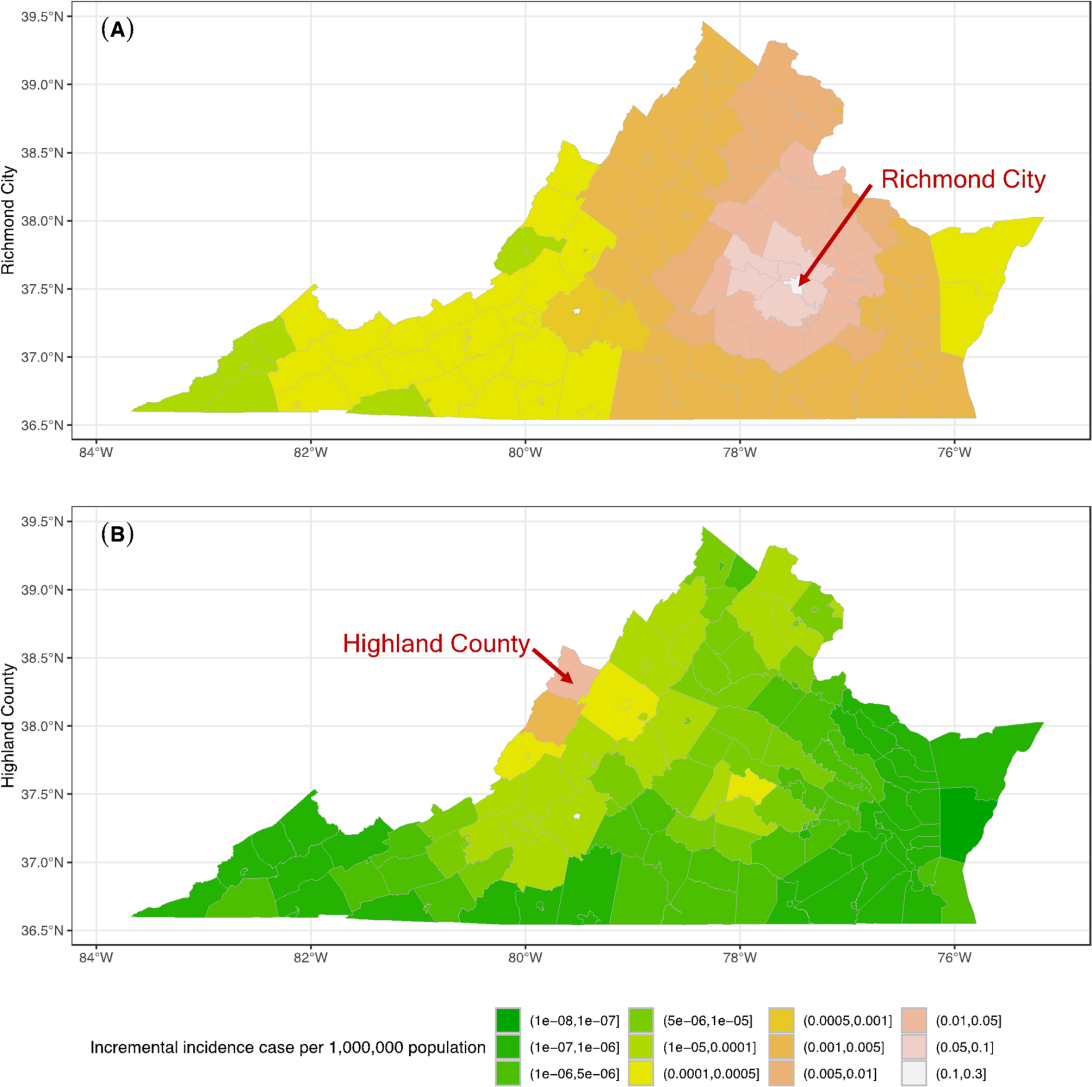
Predicted incremental incidence case per 1,000,000 population when MMR level reduced by 1% (**A**) at Richmond City, or (**B**) at Highland County as a benchmark scenario.

Under the benchmark scenario where post-COVID transmissibility is set at 0.5 and home isolation/quarantine compliance is at 75%, the model projects a discernible increase in measles cases in Richmond City and its vicinity (Fig. 6A). The most significant impact is observed in Hanover County, with a projected increase of 0.07 measles cases per 1,000,000 population. Powhatan County follows closely with an additional 0.06 cases per 1,000,000 population. Conversely, the examination of Highland County, as illustrated in Fig. 6B, reveals that spillover effects predominantly affect adjacent counties, attributable to its low population density and geographical isolation. Notably, Powhatan County experiences a modest uptick in both measles incidence and associated costs. This indirect impact occurs despite the absence of direct commuting patterns between Highland and Powhatan Counties. Instead, Bath County, situated as an intermediary and sharing commuting connections with Powhatan County, serves as the conduit for these second-order spillover effects. This phenomenon underscores the significance of indirect pathways in the transmission of measles and its economic repercussions, even in the absence of direct interactions.

**Fig. 7:**
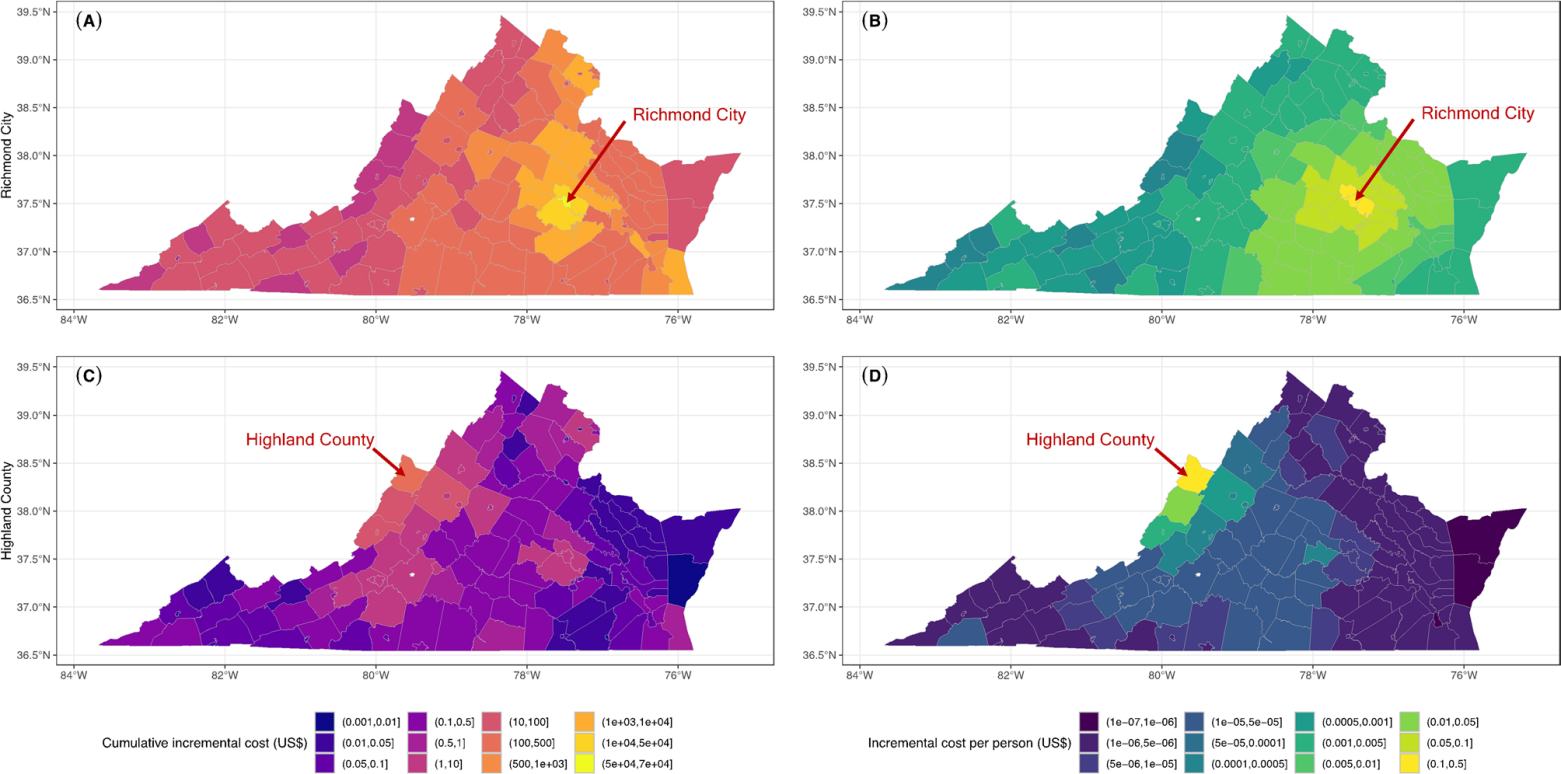
(Left) Predicted cumulative incremental cost and (Right) incremental cost per person (US$) when MMR level reduced by 1% at (Top) Richmond City and (Bottom) Highland County as a benchmark scenario, respectively.

**Fig. 8:**
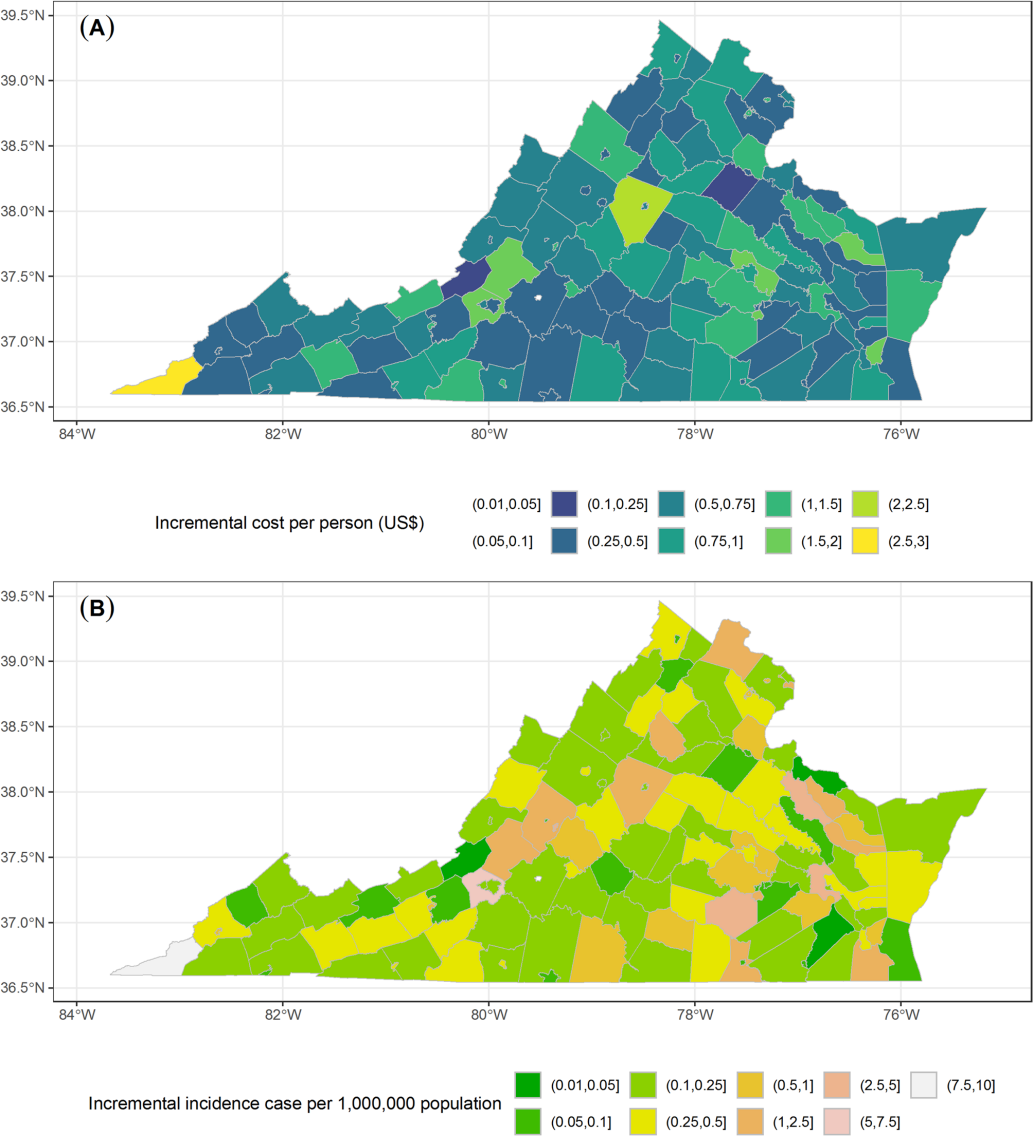
Total spillover effects of a 1% reduction in MMR vaccination rates in each Virginia county: (**A**) Total incremental costs per person (US$) and (**B**) Total incremental incidence cases per 1,000,000 population.

The economic impact in the Richmond City scenario is also noteworthy (Fig. 7A,B). Henrico County registers the top incremental cost at $34,664 in total and $0.116 per capita, while Chesterfield County encounters a rise of $29,786 ($0.096 per capita). Remarkably, certain counties, although geographically distant from Richmond City, still face notable economic burdens. For instance, Beach City, situated at Virginia’s southeast corner, incurs an added cumulative cost of $1598. This underlines the overarching interconnectedness of Virginia’s communities. Conversely, counties with a greater distance from Richmond and a considerably smaller populace like Norton City, witness almost no change in cost. The economic impact in Highland County (Fig. 7C,D) mirrors its epidemiological pattern, with minimal spillover to distant counties. The corresponding prediction intervals for the two regions are shown in the Supplementary Appendix S3 (Fig. S1–S4). We also examine the total spillover effects of a 1% decrease in MMR vaccination rate across 133 counties, which is visualized in Fig. 8.

## Discussion

This paper aims to study the health and economic consequences resulting from a reduction in MMR vaccination rate in the aftermath of the COVID-19 pandemic. Our study combines epidemiological insights with a comprehensive economic assessment. We apply a generalized spatial autoregressive (GSAR) model to assess the regional effects of a decreased MMR rate under different scenarios. This model improves upon the traditional SAR by accounting for broader spatial interactions and nonlinearities. Our findings imply that declining MMR vaccination rates have considerable health and economic repercussions, with spatial considerations playing a key role. Significant statistical differences are observed for critical variables including MMR levels, disease transmission rate, and adherence to home isolation/quarantine rates.

### Health and economic implications of reduced vaccination rates

The study highlights a dual burden: an increase in measles cases and the associated economic repercussions. The economic burden of measles outbreak ranges from direct costs related to medical and public health responses to indirect expenses such as lost productivity. We found that a decline in vaccination rate in Virginia of more than 15% would precipitate a steep rise in measles incidence and increase measles-related economic costs by orders of magnitude. Moreover, as vaccination rates decline, the effective reproduction number ${R}_{t}$ also increases and even exceeds 5 at the early stage of the outbreak, reflecting greater epidemic potential even with high home isolation compliance rates and lower transmission rates. This indicates that maintaining high vaccination rates plays a substantially larger role in constraining early epidemic growth than variations in individual behavioral compliance or baseline transmissibility within the ranges examined. Importantly, the effectiveness of home isolation as a mitigation strategy itself depends on the level of vaccination coverage. At higher vaccination coverage, the size of the susceptible pool is small, and most infections occur in small, isolated clusters, which causes transmission chains to die out quickly. Even under higher transmission rates, increased compliance with home isolation has limited impact because infectious individuals rarely encounter susceptible, and outbreaks are naturally contained. In contrast, as vaccination coverage declines, the susceptible pool grows, and transmission becomes more sustained and widespread. In such settings, compliance with home isolation becomes increasingly important, and reducing contact between infectious and susceptible individuals can slow the spread and reduce outbreak size. Therefore, in contexts where high vaccination coverage cannot be ensured, preemptive interventions at an early stage are essential. Interventions such as faster and higher adherence to home quarantine cannot completely counteract the burden of low vaccination coverage but can mitigate some loss.

It is important to note that our economic burden estimates rely on several simplifying assumptions that are commonly used in the health economics literature. First, we assume that every measles infection is reported and treated either as an inpatient or outpatient case. This may overestimate treatment costs for underinsured or marginalized populations. However, such a conservative approach is not necessarily undesirable in the context of public health planning, as it ensures that policymakers and health systems are better prepared for potential worst-case economic impacts when allocating resources for outbreak response. Additionally, while our model incorporates household-level heterogeneity through differences in household income and employment structure, it does not fully capture variation in out-of-pocket costs across individuals with different socio-economic statuses. That said, as illustrated in Fig. 5, treatment costs and productivity losses represent only a small fraction relative to vaccination costs in our scenarios, suggesting that finer individual-level adjustments would likely have a limited impact on our overall conclusions. Finally, our analysis is purposefully focused on the effects of household-level interventions—specifically, changes in vaccination coverage and home isolation compliance—and does not extend to broader societal interventions, such as school or public service closures. We highlight these factors as important areas for future work to improve the equity sensitivity and comprehensiveness of economic burden estimates.

### Spatial dependencies and spillover impact

Our simulation and GSAR model results highlight the importance of spatial relationships in shaping both measles transmission and its associated economic burden. By using inter-county commuting patterns, we capture how infections and costs extend beyond county borders through human mobility. The statistically significant spatial lag terms confirm that a county’s outcomes are influenced by what’s happening in neighboring areas—especially in places with high daily movement, such as urban and suburban regions. This spatial interdependence sets the stage for understanding how the position and connectivity of each county within the broader mobility network can amplify or buffer outbreak impacts.

We observe heterogeneous spillover effects depending on the urbanicity and network position of each county. Our GSAR model estimates show that counties with greater connectivity act as both amplifiers and transmitters of outbreak risks. In particular, urban hubs like Richmond demonstrate strong outward spatial lag effects: a localized decline in vaccination coverage leads to elevated burdens not only within the city but also across neighboring counties. This spillover is nonlinear and asymmetric—rural counties with sparse connectivity like Highland may absorb shocks from nearby cities but exert limited reciprocal influence. This asymmetry underscores a key insight: the epidemic spread of a community is shaped not solely by its own characteristics, but by its position within the spatial network of mobility.

Our results demonstrate that individual behavior—particularly adherence to home isolation—plays a crucial role in shaping how outbreaks spread across regions. When compliance is low, disease transmission intensifies and extends more readily from one area to another. Densely connected urban centers like Richmond can become sources of wider regional spread. Encouragingly, even modest improvements in isolation compliance within these highly connected hubs can significantly reduce the downstream impact on neighboring counties. This highlights the importance of tailoring public health strategies to both regional connectivity and behavioral dynamics, rather than applying uniform measures across all areas.

From a policy perspective, these insights point to the value of targeted interventions over blanket approaches. Prioritizing efforts such as MMR catch-up vaccinations or stricter isolation enforcement in central, well-connected counties can yield outsized benefits statewide. By contrast, applying the same resources in more isolated or low-connectivity regions may result in more limited, localized impacts. In this context, spatial dependency is not merely a technical detail in modeling—it offers actionable guidance for optimizing resource allocation and maximizing public health outcomes.

### Limitations

However, this study is subject to certain limitations in terms of the agent-based model. First, the model assumes 100% vaccine effectiveness. Nevertheless, the existing academic literature^40–42^ have documented occurrences of measles infections among individuals who have had vaccinations, and it is important to note that these cases typically exhibit less severe symptoms and are less likely to result in secondary transmissions^43^. Future work could incorporate a leaky vaccine framework to account for partial protection and breakthrough infections. Second, the model assumes a return to pre-pandemic levels of mobility and social mixing, which may not fully reflect ongoing behavioral shifts in the post-COVID era^44,45^. Third, the simulation is initialized with a randomly selected individual, aged 5–17 years, as the index case, following previous studies. While this school age group was chosen because it is likely to have high contact rates and hence a high probability of an outbreak, results may vary if the index case is drawn from a different age group. Fourth, this model assumes that the decline in MMR vaccination coverage is uniformly distributed across the population in Virginia. Previous work by Thakur et al. (2022)^6^ explored alternative vaccination decline distributions, including scenarios weighted by per capita household income to simulate greater impact on low-income or vulnerable populations. In our study, we adopt the uniform decline scenario as a representative case to demonstrate the framework. However, our methodology is flexible and can be extended to incorporate alternative, more realistic distributional assumptions in future analyses.

The GSAR model also has its own limitations. The confounding variables used here may not be comprehensive, perhaps other factors such as environmental influences, cultural differences, or region-specific occurrences may have significant effects. Furthermore, although our spatial analysis is extensive, it does not further explore the possibility of a more advanced spatial model. This leaves open opportunities for future research. For example, the spatial weight matrix can be modeled as a function of the commute flow matrix and the distance matrix; or a more complicated model such as generalized spatial autoregressive combined model (SAC) or generalized spatial Durbin model (SDM) can be considered. While more complex models offer deeper insights, our choice of a simpler spatial analysis ensures clarity, interpretability, and ease of replication in studying foundational patterns, as explained in the Supplementary Appendix S2.3 (Tables S4, S5). In addition, the geographic scope of this study is limited to Virginia.

## Conclusions

This research studies the health and economic implications of reduced MMR vaccination rates in Virginia. We use a scenario-based analysis to quantify the epidemiologic and economic burden of potential measles flare-ups, locally, as well as in the neighboring regions caused by spatial spillovers. A Generalized Spatial Autoregressive Model that incorporates commuting flow data from the US census, is used to estimate the ‘spillover effects’ of reduced MMR rates and to understand the differential impact of reduced MMR rates in rural versus urban regions.

Our findings indicate significant spatial interdependencies. A small decrease in MMR vaccination rate in Richmond City, an urban region, prompts marked spillover in adjacent areas and even statewide, while similar reductions in Highland County, a rural area, produce a limited spillover effect.

This study highlights the crucial need of implementing evidence-based policymaking that aligns with health and economic goals. When developing and carrying out public health interventions like vaccination campaigns and targeted surveillance programs, officials must consider the spatial interdependence between regions as they have broader health and economic consequences.

## Supplementary Information

Below is the link to the electronic supplementary material.

Supplementary Material 1

## Data Availability

The code and associated datasets have been made publicly accessible via GitHub: https://github.com/xkx842044566/SpatialSpilloverEffect_Measles. The repository contains all scripts used for data processing, figure generation, GSAR modeling, and spillover effects estimation. Due to file size limitations, we have provided the aggregated simulation outputs for measles incidence and cost analyses used in the manuscript. Researchers interested in obtaining the full agent-based simulation results are encouraged to contact Kexin Xie (kexinx@vt.edu) directly.

## Abbreviations

MMR: Measles, Mumps and Rubella
INLA: Integrated nested laplace approximations
SAR: Spatial autoregressive model
GSAR: Generalized spatial autoregressive model
GLM: Generalized linear model
HIQ_Cγ: Home isolation/quarantine compliance
HI_D: Home isolation delay
HQ_D: Home quarantine of delay
HI_P: Home isolation adherence probability
MMR_E: Effectiveness of MMR vaccine
HI_T: Duration of home isolation
HQ_T: Duration of home quarantine
TR$\tau$: Transmissibility rate
MMRα: MMR level reduction
VA: Virginia
SEIR: Susceptible-exposed-infected-recovered
USD: US dollars
CDC: Center for Disease control and prevention
DIC: Deviance information criterion
WAIC: Watanabe-Akaike Information Criterion
LOOCV: Leave-one-out cross-validation
PRMSE: Predictive root mean square error
SAC: Spatial autoregressive combined model
SDM: Spatial Durbin model
