# Supplementary material for: A health and economic evaluation of the spatial spillover effect from measles resurgence
Source: Sci Rep. 2025 Oct 14;15:35862. doi: 10.1038/s41598-025-21097-0 (PMC12521593; doi:10.1038/s41598-025-21097-0)
Supplement: Supplementary file 1 — Supplementary Material 1 [file 41598_2025_21097_MOESM1_ESM.pdf]

Supplementary Material

Supplementary Material *1*

1. Methodology 2
   1.1 Marginal Effects in GSAR 2
2. Robust Analysis 2
   2.1 Summary statistics of measles cost 2
   Table S1. Summary statistics (mean and standard deviation) of the MMR vaccination cost, treatment cost, productivity loss and total cost (in million US$), stratified by scenarios. 2
   2.2 Spatial Diagnose 3
   Table S2. The BSK test results for spatial autoregression and random effects. 3
   Table S3. The LM test results for spatial lag dependency and spatial error dependency. 4
   2.3 Alternative Spatial Specifications 4
   Table S4. The Estimation Results on Measles Incidence Cases Using Alternative Poisson Spatial Models. Numbers in bold reflect that the parameter estimation is significant at the 95% confidence level. 4
   Table S5. The Estimation Results on Measles Cost per Capita Using Alternative Gamma Spatial Models. Numbers in bold reflect that the parameter estimation is significant at the 95% confidence level. 5
   2.4 Endogeneity of lagged covariates 6
3. Predictive Interval 7
   Figure S1. (A) Lower bound (2.5%) of predictive interval for the incremental incidence case per 1,000,000 population when MMR level reduced by 1% (A) at Richmond City, or (B) at Highland County as a benchmark scenario. 7
   Figure S2. (A) Upper bound (97.5%) of predictive interval for the incremental incidence case per 1,000,000 population when MMR level reduced by 1% (A) at Richmond City, or (B) at Highland County as a benchmark scenario. 8
   Figure S3. (A) (Left) Lower bound (2.5%) of predictive interval for the cumulative incremental cost and (Right) incremental cost per person (US$) when MMR level reduced by 1% at (Top) Richmond City and (Bottom) Highland County as a benchmark scenario, respectively. 9
   Figure S4. (A) (Left) Upper bound (97.5%) of predictive interval for the cumulative incremental cost and (Right) incremental cost per person (US$) when MMR level reduced by 1% at (Top) Richmond City and (Bottom) Highland County as a benchmark scenario, respectively. 9
4. Uncertainty analysis 10
   4.1 Marginal effects from Generalized-SAR model. 10
   Table S6. Marginal Direct Impacts, Indirect (Spillover) Impacts and Total Impacts for (A) 90th percentile Measles Cases derived from GSAR-Poisson Model, and (B) 90th percentile Measles Costs Per Capita derived from GSAR-Gamma Model. Numbers in bold reflect that the parameter estimation is significant at the 95% confidence level. 11
   4.2 Prediction results 11
   Figure S5. (A)Predicted 90th percentile incremental incidence case per 1,000,000 population when MMR level reduced by 1% (A) at Richmond City, or (B) at Highland County as a benchmark scenario. 12
   Figure S6. (Left) Predicted 90th percentile cumulative incremental cost and (Right) incremental cost per person (US$) when MMR level reduced by 1% at (Top) Richmond City and (Bottom) Highland County as a benchmark scenario, respectively. 13
5. References 13

1. **Methodology**

1.1 Marginal Effects in GSAR

To compute the direct and spillover effects in GSAR (say, $g(\cdot)$=log($\cdot$)), it is necessary to decompose the model in the following reduced form:

$$\log(\boldsymbol{\mu}) = \boldsymbol{\eta} = (\boldsymbol{I_n} - \rho \boldsymbol{W})^{-1} \boldsymbol{X\beta}, \quad (1)$$

$$\Rightarrow E(\boldsymbol{y}) = \boldsymbol{\mu} = \exp[(\boldsymbol{I_n} - \rho \boldsymbol{W})^{-1} \boldsymbol{X\beta}], \quad (2)$$

$$\Rightarrow E(\boldsymbol{y}) = \boldsymbol{\mu} = \exp\left[\sum_{k=1}^{p} (\boldsymbol{I_n} - \rho \boldsymbol{W})^{-1} \beta_k \boldsymbol{x_k}\right], \quad (3)$$

By considering the matrix of partial derivatives pertaining to the expected value of $\boldsymbol{y}$, $E(\boldsymbol{y})$, with respect to the kth covariate of X across units 1 to s, it is formulated as:

$$\frac{\partial E(\boldsymbol{y})}{\partial \boldsymbol{x_k}^T} = \left[\frac{\partial E(\boldsymbol{y})}{\partial x_{1k}}, \frac{\partial E(\boldsymbol{y})}{\partial x_{2k}}, \dots, \frac{\partial E(\boldsymbol{y})}{\partial x_{Sk}}\right] = D(f(\boldsymbol{\eta}))(\boldsymbol{I_n} - \rho \boldsymbol{W})^{-1} \beta_k. \quad (4)$$

Here $f(\boldsymbol{\eta})$ denotes a vector comprising the exponential transformations of the linear predictors $\eta_i, i = 1, \dots, n$. The notation $D(\cdot)$ represents a $n \times n$ diagonal matrix derived from its argument. The right side of (4) is denoted as $S_k(W)$, where $S_k(W)_{ij}$ is its i,jth element.

A better comprehension of direct and spillover effects can be achieved through an examination of the Taylor series expansion of the spatial multiplier matrix:

$$(\boldsymbol{I_n} - \rho \boldsymbol{W})^{-1} = \boldsymbol{I_n} + \rho \boldsymbol{W} + \rho^2 \boldsymbol{W}^2 + \cdots \quad (5)$$

In formulation (5), each exponentiation of $\boldsymbol{W}$ signifies varying degrees of spatial interaction: the zero-order reflects the observations themselves, the first-order represents immediate neighbors, the second-order encompasses neighbors of neighbors, and so forth. By employing this series expansion, we can estimate not only the cumulative effects but also indirect effects attributed to distinct orders of spatial proximity [1].

Regarding the impact assessment, the diagonal elements of $S_k(W)$ represent direct effects, while the off-diagonal elements consist of the spillover effects of the kth covariate. As defined by LeSage and Pace (2009) [1], different average impacts of the kth covariate are computed as follows:

$$\bar{M}(k)_{direct} = \frac{1}{n} tr(S_k(W)), \quad (6)$$

$$\bar{M}(k)_{total} = \frac{1}{n} \boldsymbol{l}' S_k(W) \boldsymbol{l}, \quad (7)$$

$$\bar{M}(k)_{indirect} = \bar{M}(k)_{total} - \bar{M}(k)_{direct.} \quad (8)$$

However, due to multi-parameters and non-linear terms, these effects cannot be straightforwardly computed via the linear combinations of β_k. Thus, we approximate the posterior marginal of the average impacts by sampling from the approximate joint posterior in INLA, as proposed by Goméz-Rubio et al. (2020, Chapter 2)[2].

2. **Robust Analysis**

2.1 Summary statistics of measles cost

Table S1. Summary statistics (mean and standard deviation) of the MMR vaccination cost, treatment cost, productivity loss and total cost (in million US$), stratified by scenarios.

	Transmissibility rate $\tau = 0.4$			Transmissibility rate $\tau = 0.5$			Transmissibility rate $\tau = 0.6$		
	$\gamma = 75$	$\gamma = 85$	$\gamma = 95$	$\gamma = 75$	$\gamma = 85$	$\gamma = 95$	$\gamma = 75$	$\gamma = 85$	$\gamma = 95$
MMR level reduction % ($\alpha = 0$)									
MMR cost	-	-	-	1603.81	1603.81	1603.81	-	-	-
Treatment cost	-	-	-	2.84 (9.93)	2.01 (7.84)	0.79 (3.46)	-	-	-

Productivity loss cost	-	-	-	2.32 (8.40)	2.01 (8.10)	0.96 (4.08)	-	-	-
Total cost	-	-	-	1608.97 (18.31)	1607.83 (15.93)	1605.56 (7.52)	-	-	-
MMR level reduction % ($\alpha = 5$)									
MMR cost	1584.22	1584.22	1584.22	1584.22	1584.22	1584.22	1584.22	1584.22	1584.22
Treatment cost	1.14 (8.02)	0.80 (5.68)	0.12 (0.73)	6.81 (27.33)	2.97 (15.25)	1.27 (7.49)	15.48 (48.34)	5.00 (22.57)	4.57 (19.95)
Productivity loss cost	0.93 (6.81)	0.82 (6.03)	0.13 (0.67)	5.73 (24.49)	2.95 (16.11)	1.60 (9.48)	12.59 (41.44)	4.63 (21.70)	5.22 (23.62)
Total cost	1586.29 (14.81)	1585.84 (11.70)	1584.47 (1.39)	1596.77 (51.75)	1590.14 (31.31)	1587.10 (16.95)	1612.30 (89.68)	1593.85 (44.22)	1594.02 (43.52)
MMR level reduction % ($\alpha = 15$)									
MMR cost	1545.05	1545.05	1545.05	1545.05	1545.05	1545.05	1545.05	1545.05	1545.05
Treatment cost	9.85 (43.09)	7.13 (34.83)	1.97 (13.43)	34.61 (96.26)	15.28 (62.02)	11.52 (50.29)	120.71 (235.50)	54.01 (139.38)	29.64 (95.58)
Productivity loss cost	6.98 (34.29)	6.61 (36.77)	2.12 (16.96)	23.47 (13.27)	13.27 (65.64)	12.81 (62.57)	94.63 (48.40)	48.40 (146.73)	33.01 (123.84)
Total cost	1561.88 (77.22)	1558.80 (71.48)	1549.15 (30.34)	1603.14 (173.54)	1573.60 (127.11)	1569.39 (112.62)	1760.39 (442.41)	1647.46 (285.03)	1607.71 (218.90)
MMR level reduction % ($\alpha = 25$)									
MMR cost	1505.88	1505.88	1505.88	1505.88	1505.88	1505.88	1505.88	1505.88	1505.88
Treatment cost	258.36 (368.61)	144.02 (269.24)	82.01 (193.61)	598.31 (574.27)	365.44 (476.34)	200.19 (342.10)	891.24 (803.32)	674.82 (687.25)	344.29 (478.57)
Productivity loss cost	202.37 (332.05)	137.69 (295.67)	94.73 (252.96)	455.73 (490.51)	342.19 (504.11)	232.53 (450.60)	513.20 (633.05)	167.23 (448.83)	388.16 (604.92)
Total cost	1966.62 (697.34)	1787.59 (562.85)	1682.62 (445.37)	2559.93 (1059.19)	2213.51 (976.42)	1938.61 (789.94)	2910.33 (1350.43)	2347.94 (958.22)	2238.34 (1079.65)

2.2 Spatial Diagnose

From the 9,000 simulation experiments, we gathered comprehensive data detailing measles incidence, isolation/quarantine duration, MMR vaccination expenditures, medical treatment costs, and productivity losses. For spatial analysis, the simulated incidence and cost outputs were consolidated by county, for all 133 counties in Virginia, under 30 distinct experimental scenarios, forming a panel dataset. Given the inclusion of experimental settings as predictors in our spatial econometric model and the consequent absence of within-group variation, a fixed effects specification was deemed inapplicable. Based on the Gaussian linear model fitted to the log of cost per capita and log of incidence, we employed the Baltagi and Song [3] methodology (BSK test) to test for spatial autoregression and random effects within the panel framework. The results, presented in Table S2, substantiate the presence of spatial autoregression without random effects, suggesting the adoption of a pooled spatial autoregressive model for this analysis.

Table S2. The BSK test results for spatial autoregression and random effects.

Null Hypothesis	Alternative Hypothesis	Tests results for Log-cost per capita	Tests results for Log-incidence

Spatial autoregression and random effect are nonexistent	Spatial autoregression or random effect are existent	Statistics = 36.3 (p-value = 4.124e-09)	102.82 (p-value < 2.2e-16)
Random effect is nonexistent	Random effect is existent	Statistics = -0.448 (p-value = 1.346)	0.884 (p-value = 1.041)
Spatial autoregression is nonexistent	Spatial autoregression was existent	Statistics = 6.0249 (p-value = 1.692e-09)	8.887 (p-value < 2.2e-16)

Subsequent Lagrange Multiplier diagnostics [4], detailed in Table S3, revealed that the non-spatial model for costs and the Spatial Autoregressive Model (SAR) for incidence rates were statistically preferred. However, the Spatial Autoregressive (SAR) model was selected for cost model. This is determined not only by the outcome of the BSK test, but also by the advantageous interpretability of the SAR model in the context of our research objectives. The SAR model allows for a nuanced delineation of direct and indirect economic impact attributable to measles resurgence, which aligns with our goal to elucidate the broader economic ramifications of the disease's propagation.

Table S3. The LM test results for spatial lag dependency and spatial error dependency.

Lagrange Multiplier Test	Tests results for Log-cost per capita	Tests results for Log-incidence
Spatial error		
LM	0.144 (p-value = 0.704)	2.775 (p-value = 0.096)
LM robust	3.609 (p-value = 0.058)	0.877 (p-value = 0.349)
Spatial lag		
LM	1.699 (p-value = 0.193)	11.403 (p-value = 0.001)
LM robust	5.164 (p-value = 0.023)	9.506 (p-value = 0.002)

2.3 Alternative Spatial Specifications

The results in Table S4 and Table S5 underscore the robustness and justify the choice of the GSAR model. The results of GSAR yield a consistent intercept and coefficients for key variables, including MMR level reduction rate, transmissibility, and home isolation/quarantine compliance, across all spatial model variations. While the SDEM-Poisson and SEM-Gamma provided marginally better fits according to DIC and WAIC criteria, the GSAR model yielded a lower RMSE value for both cases. This suggests a more parsimonious explanation of the data's variance, validating our choice in the context of interpreting direct and indirect effects. The Moran's I statistics across models signals spatial independence of residuals, validating our GSAR model selection and its capacity to capture spatial interdependencies adequately without residual autocorrelation. The robustness of these results across alternative spatial specifications underscores the GSAR model's capacity to capture the complex interdependencies inherent in our analysis.

Table S4. The Estimation Results on Measles Incidence Cases Using Alternative Poisson Spatial Models. Numbers in bold reflect that the parameter estimation is significant at the 95% confidence level.

Variables	SAR-Poisson	SEM-Poisson	SDM-Poisson	SDEM-Poisson
	Coef. (Std. Err.)	Coef. (Std. Err.)	Coef. (Std. Err.)	Coef. (Std. Err.)
(Intercept)	**-3.983 (-4.289, -3.708)**	**-14.189 (-14.569, -13.816)**	**-4.205 (-4.534, -3.883)**	**-14.388 (-14.721, -14.058)**
MMR level reduction % (α)	**0.090 (0.082, 0.097)**	**0.322 (0.306, 0.339)**	**0.094 (0.086, 0.102)**	**0.328 (0.314, 0.342)**
Transmissibility (τ)[1]				

[1] The reference level of transmissibility (τ) is 0.5.

0.4 (Low level)	**-0.382 (-0.466, -0.300)**	**-1.399 (-1.728, -1.071)**	**-0.402 (-0.486, -0.318)**	**-1.409 (-1.687, -1.131)**
0.6 (High level)	**0.281 (0.207, 0.354)**	**0.996 (0.695, 1.298)**	**0.293 (0.219, 0.367)**	**1.013 (0.760, 1.267)**
Home isolation/quarantine compliance % (γ)[2]				
85 (Median level)	**-0.195 (-0.269, -0.121)**	**-0.708 (-1.015, -0.401)**	**-0.204 (-0.278, -0.130)**	**-0.713 (-0.971, -0.454)**
95 (High level)	**-0.434 (-0.516, -0.353)**	**-1.558 (-1.875, -1.240)**	**-0.455 (-0.538, -0.373)**	**-1.584 (-1.852, -1.316)**
Average household annual income (in thousand US$)	**0.060 (0.008, 0.113)**	0.080 (-0.004, 0.165)	-0.025 (-0.113, 0.064)	-0.035 (-0.122, 0.052)
Proportion of male	**0.203 (0.163, 0.243)**	**0.219 (0.170, 0.268)**	**0.247 (0.198, 0.297)**	**0.293 (0.241, 0.345)**
Proportion of children under 5 years old	**0.085 (0.035, 0.136)**	**0.222 (0.153, 0.291)**	**0.223 (0.152, 0.293)**	**0.229 (0.160, 0.299)**
Proportion of employed population	**-0.212 (-0.281, -0.143)**	**-0.246 (-0.349, -0.144)**	-0.016 (-0.124, 0.092)	-0.036 (-0.142, 0.071)
LAG: Average household annual income (in K US$)			0.003 (-0.014, 0.019)	**0.308 (0.142, 0.475)**
LAG: Proportion of male			0.003 (-0.006, 0.012)	**-0.137 (-0.243, -0.032)**
LAG: Proportion of children under 5 years old			-0.009 (-0.022, 0.004)	**-0.259 (-0.391, -0.127)**
LAG: Proportion of employed population			-0.005 (-0.025, 0.014)	**-0.846 (-1.050, -0.642)**
DIC	8377.86	8363.61	8365.80	8323.80
WAIC	8108.16	8088.53	8086.47	8038.85
RMSE	0.773	0.776	0.765	0.774
Moran's I statistics	-0.235	-0.246	-0.230	-0.234
Response Lag coefficient (ρ)	**0.717 (0.696, 0.736)**		**0.702 (0.680, 0.725)**	
Error lag coefficient (λ)		**0.768 (0.749, 0.786)**		**0.719 (0.695, 0.742)**

Table S5. The Estimation Results on Measles Cost per Capita Using Alternative Gamma Spatial Models. Numbers in bold reflect that the parameter estimation is significant at the 95% confidence level.

Variables	SAR-Gamma	SEM-Gamma	SDM-Gamma	SDEM-Gamma
	Coef. (Std. Err.)	Coef. (Std. Err.)	Coef. (Std. Err.)	Coef. (Std. Err.)
(Intercept)	**1.866 (1.854, 1.867)**	**5.343 (5.320, 5.366)**	**2.636 (2.617, 2.673)**	**5.535 (5.312, 5.359)**
MMR level reduction % (α)	**0.001 (0.001, 0.001)**	**0.003 (0.002, 0.004)**	**0.002 (0.002, 0.002)**	**0.005 (0.004, 0.006)**
Transmissibility (τ)[3]				

[2] The reference level of home isolation/quarantine compliance % (γ) is 75.

[3] The reference level of transmissibility (τ) is 0.5.

0.4 (Low level)	**-0.012 (-0.019, -0.005)**	**-0.038 (-0.061, -0.061)**	**-0.020 (-0.030, -0.010)**	**-0.046 (-0.068, -0.023)**
0.6 (High level)	**0.006 (0.000, 0.003)**	-0.020 (-0.002, 0.043)	**0.010 (0.000, 0.020)**	**0.023 (0.000, 0.045)**
Home isolation/quarantine compliance % (γ)[4]				
85 (Median level)	**-0.008 (-0.014, -0.001)**	**-0.024 (-0.046, -0.001)**	**-0.012 (-0.022, -0.002)**	**-0.028 (-0.050, -0.005)**
95 (High level)	**-0.012 (-0.019, -0.005)**	**-0.039 (-0.008, -0.014)**	**-0.020 (-0.030, -0.010)**	**-0.045 (-0.067, -0.022)**
Average household annual income (in thousand US$)	**0.007 (0.002, 0.012)**	**0.013 (0.006, 0.020)**	0.009 (-0.004, 0.022)	0.010 (-0.002, 0.023)
Proportion of male	**0.005 (0.002, 0.008)**	**0.003 (0.000, 0.007)**	0.005 (-0.001, 0.011)	**0.007 (0.001, 0.013)**
Proportion of children under 5 years old	**0.007 (0.003, 0.011)**	**0.010 (0.004, 0.015)**	**0.012 (0.002, 0.022)**	**0.012 (0.002, 0.021)**
Proportion of employed population	**-0.006 (-0.011, 0.000)**	**-0.010 (-0.018, -0.003)**	-0.004 (-0.020, 0.011)	-0.005 (-0.020, 0.009)
LAG: Average household annual income (in K US$)			0.003 (-0.014, 0.019)	0.014 (-0.006, 0.034)
LAG: Proportion of male			0.003 (-0.006, 0.012)	0.005 (-0.007, 0.016)
LAG: Proportion of children under 5 years old			-0.009 (-0.022, 0.004)	-0.002 (-0.017, 0.013)
LAG: Proportion of employed population			-0.005 (-0.025, 0.014)	-0.014 (-0.037, 0.010)
DIC	11489	11481.48	37003.45	37001.96
WAIC	10695	10691.67	35753.15	35751.81
RMSE	0.019	0.020	8.062	8.045
Moran's I statistics	-0.215	-0.219	0.227	0.218
Response Lag coefficient (ρ)	**0.686 (0.667, 0.703)**		**0.562 (0.533, 0.590)**	
Error lag coefficient (λ)		**0.698 (0.679, 0.715)**		**0.566 (0.537, 0.595)**

2.4 Endogeneity of lagged covariates

In our simulation-based GSAR model analysis, the systematic variation of parameters—MMR level reduction % (α), transmissibility (τ), and home isolation/quarantine compliance % (γ)—allows us to observe their direct influence on economic outcomes without the traditional endogeneity concerns present in observational studies. Endogeneity, typically addressed in empirical research through instruments or alternative specifications, is inherently controlled in our simulation design.

Furthermore, the application of GSDM and GSDEM serves to scrutinize the potential endogeneity in lagged covariates. These models, showing insignificant spatially lagged independent variables, provide a robust support to verify the absence of endogenous bias in our simulated data. The consistency of our findings across GSAR, GSDM, and GSDEM, reinforced by diagnostic checks such as the DIC, WAIC, and Moran's I statistics, validates the robustness of our model and the reliability of the causal inferences drawn from the simulated data.

[4] The reference level of home isolation/quarantine compliance % (γ) is 75.

3. Predictive Interval

In this section, we present the 95% predictive intervals for the incremental measles incidence case (Figure S1 & Figure S2), incremental cost and incremental cost per person (US$) (Figure S3 & Figure S4) when MMR level reduced by 1% in Richmond city or in Highland County as the benchmark scenarios.

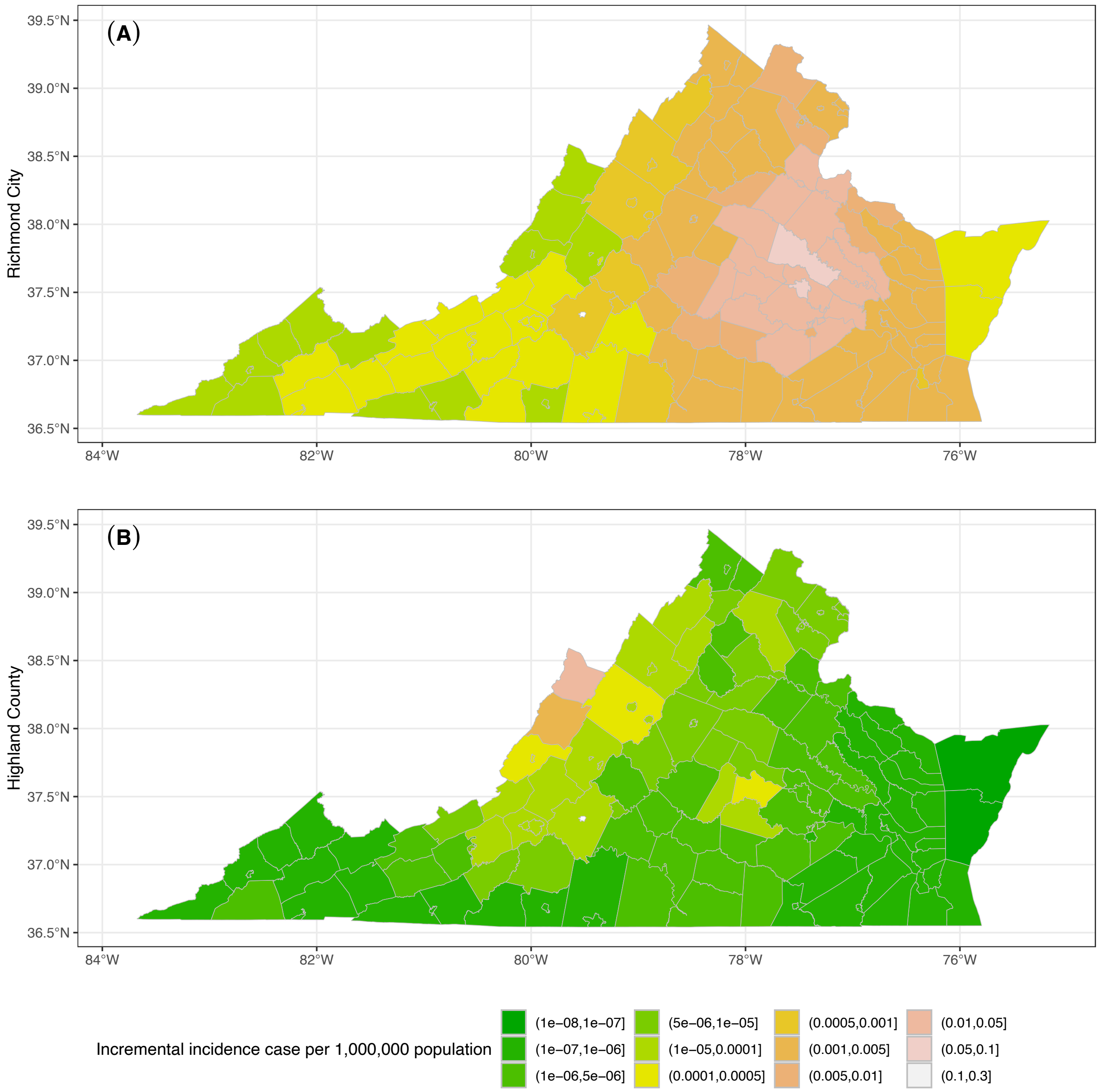

Figure S1. (A) Lower bound (2.5%) of predictive interval for the incremental incidence case per 1,000,000 population when MMR level reduced by 1% (A) at Richmond City, or (B) at Highland County as a benchmark scenario.

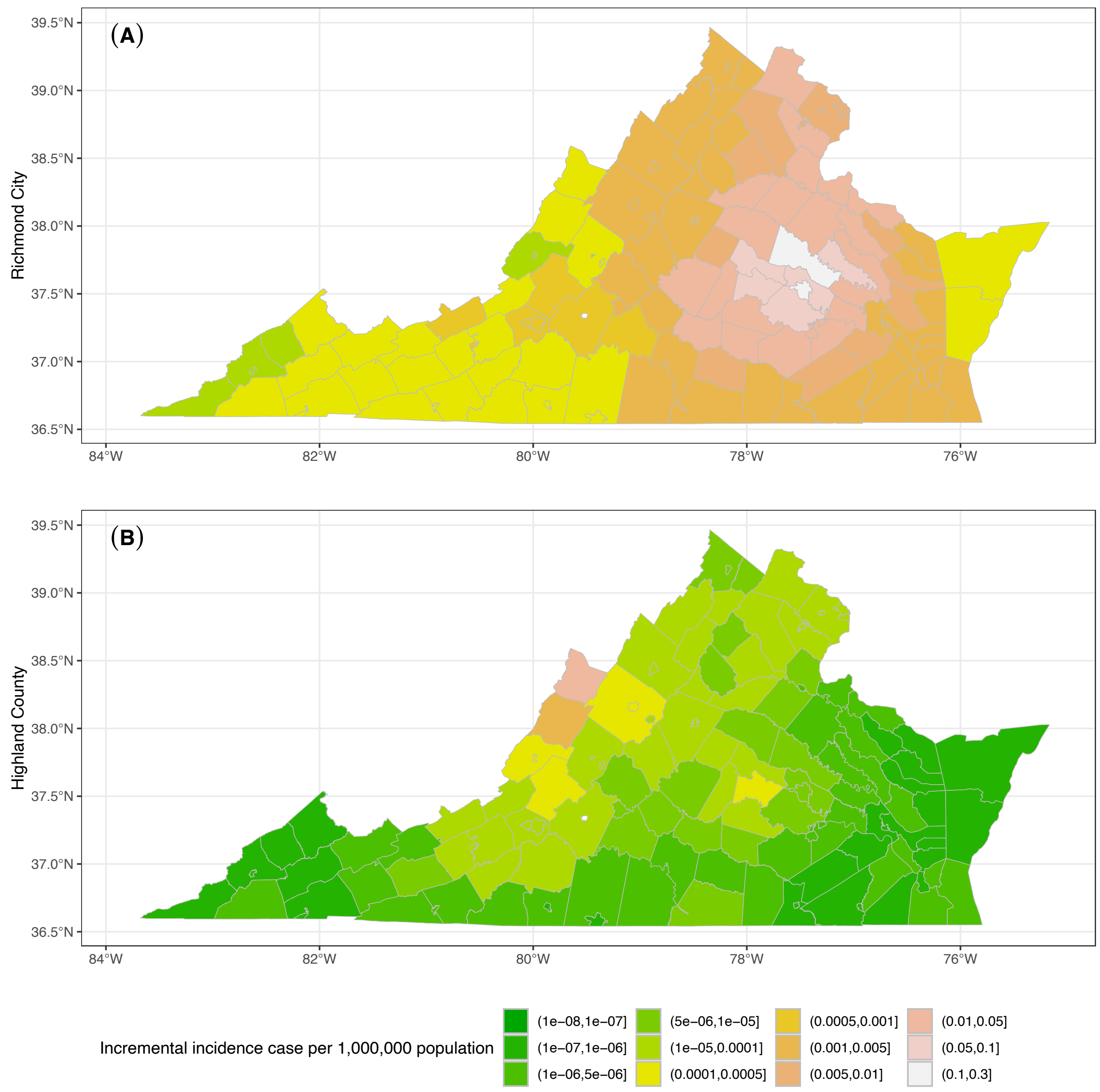

Figure S2. (A) Upper bound (97.5%) of predictive interval for the incremental incidence case per 1,000,000 population when MMR level reduced by 1% (A) at Richmond City, or (B) at Highland County as a benchmark scenario.

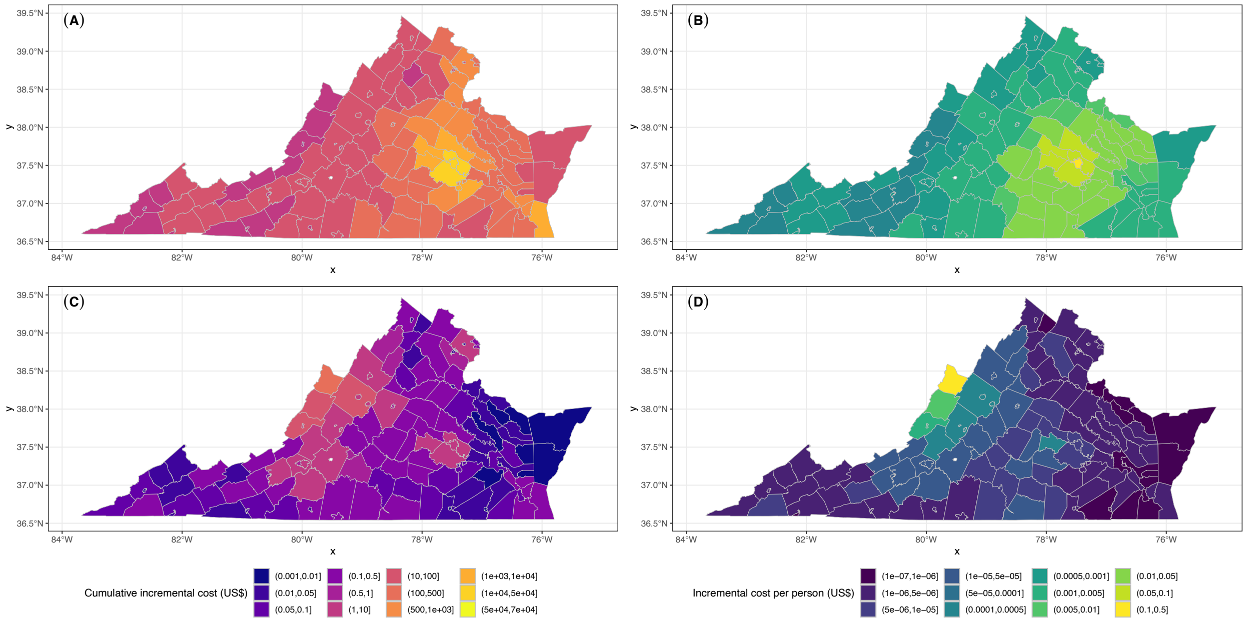

Figure S3. (A) (Left) Lower bound (2.5%) of predictive interval for the cumulative incremental cost and (Right) incremental cost per person (US$) when MMR level reduced by 1% at (Top) Richmond City and (Bottom) Highland County as a benchmark scenario, respectively.

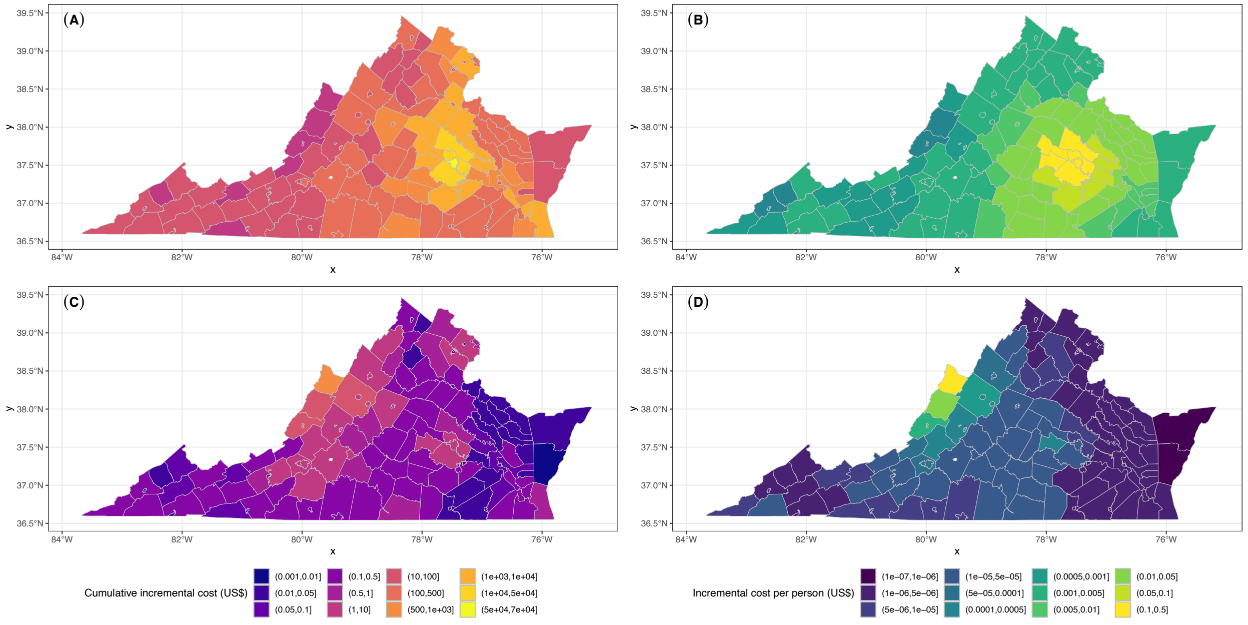

Figure S4. (A) (Left) Upper bound (97.5%) of predictive interval for the cumulative incremental cost and (Right) incremental cost per person (US$) when MMR level reduced by 1% at (Top) Richmond City and (Bottom) Highland County as a benchmark scenario, respectively.

4. Uncertainty analysis

Another unique aspect of our study is examining the uncertainty of health and economic impact. While average outcomes across states is important, the variability in health and economic burden is crucial for policy and societal development. This variability, influenced by factors like local healthcare infrastructure and regional economic dependencies, reflect the unpredictable fluctuation in health-economic burden from potential outbreaks. Our analysis seeks to understand this uncertainty, offering a detailed view of potential regional epidemiological and economic challenges. Thus, we evaluate the 90th percentile of measles cases and total costs per capita as the response variable in the Generalized-SAR model.

The motivation for the uncertainty analysis stems from the conspicuous variance observed in the simulation outcomes. The purpose of this 90th percentile modeling was to enhance comprehension regarding the stability or volatility of these outcomes across different counties. The presence of substantial fluctuation in some areas increases the likelihood of experiencing financial strain from measles. Consequently, these locations may need specific policy interventions that differ from those implemented in areas with continuously high projected average cases or costs. Following the spatial model, a comprehensive spillover analysis is then undertaken.

4.1 Marginal effects from Generalized-SAR model.

Within the 90th percentile model of the measles incidence and total cost per capita, parameters such as the decrease in MMR level, transmissibility rate, and compliance with household isolation/quarantine protocols align with the statistical significance discerned in the mean model. Likewise, coefficients associated with demographics exhibit trends analogous to those observed in the mean model. Notably, the 90th percentile in measles incidence and total costs also show spatial interdependence, as confirmed by the lag coefficient in the Generalized-SAR model ($\rho = 0.763, p < 2.22e - 16$ for measles incidence in Poisson-SAR; $\rho = 0.785, p < 2.22e - 16$ for measles total costs per capita in Gamma-SAR).

Table S6. Marginal Direct Impacts, Indirect (Spillover) Impacts and Total Impacts for (A) 90th percentile Measles Cases derived from GSAR-Poisson Model, and (B) 90th percentile Measles Costs Per Capita derived from GSAR-Gamma Model. Numbers in bold reflect that the parameter estimation is significant at the 95% confidence level.

	(A) Measles cases -- GSAR-Poisson			(B) Measles costs per capita -- GSAR-Gamma		
	Direct impact (95% CI) $\times 10^{-3}$	Indirect impact (95% CI) $\times 10^{-3}$	Total impact (95% CI) $\times 10^{-3}$	Direct impact (95% CI)	Indirect impact (95% CI)	Total impact (95% CI)
MMR level reduction % (α)	**0.48 (0.43, 0.52)**	**1.27 (1.18, 1.37)**	**1.75 (1.63, 1.86)**	**0.65 (0.49, 0.80)**	**1.55 (1.20, 1.90)**	**2.20 (1.69, 2.69)**
Transmissibility (τ)						
0.4 (Low level)	**-2.19 (-2.81, -1.56)**	**-5.82 (-7.50, -4.71)**	**-8.02 (-1.03, -5.74)**	**-5.43 (-8.70, -2.24)**	**-13.02 (-21.06, -5.38)**	**-18.45 (-29.87, -7.53)**
0.6 (High level)	**1.60 (1.01, 2.18)**	**4.24 (2.70, 5.75)**	**5.84 (3.73, 7.93)**	2.96 (-0.39, 6.02)	7.11 (-0.94, 14.49)	10.08 (-1.33, 20.52)
Home isolation/quarantine compliance % (γ)						
85 (Median level)	**-1.09 (-1.64, -0.52)**	**-2.89 (-4.33, -1.41)**	**--3.98 (-5.96, -1.94)**	**-3.57 (-7.04, -0.02)**	**-8.56 (-16.89, -0.05)**	**-12.13 (-23.84, -0.07)**
95 (High level)	**-2.38 (-2.99, -1.75)**	**-6.31 (-7.91, -4.74)**	**-8.69 (-1.08, -6.52)**	**-5.50 (-8.76, -2.07)**	**-13.20 (-20.96, -5.10)**	**-18.70 (-29.45, -7.18)**
Average household annual income (in thousand US$)	-0.16 (-0.58, 0.27)	-0.42 (-1.54, 0.71)	-0.58 (-2.11, 0.98)	**3.34 (1.06, 5.59)**	**8.03 (2.54, 13.49)**	**11.37 (3.61, 19.11)**
Proportion of male	**1.39 (1.08, 1.69)**	**3.69 (2.84, 4.63)**	**5.08 (3.96, 6.28)**	**3.27 (1.79, 4.68)**	**7.87 (4.31, 11.29)**	**11.14 (6.10, 15.91)**
Proportion of children under 5 years old	**0.45 (0.08, 0.87)**	**1.19 (0.20, 2.31)**	**1.64 (0.27, 3.16)**	**4.24 (2.39, 6.17)**	**10.20 (5.75, 14.95)**	**14.44 (8.21, 21.23)**
Proportion of employed population	**-1.56 (-2.11, -0.97)**	**-4.13 (-5.65, -2.64)**	**-5.69 (-7.66, -3.62)**	**-3.70 (-6.52, -0.85)**	**-8.88 (-15.78, -2.02)**	**-12.57 (-22.28, -2.89)**

4.2 Prediction results

We further examine the 90th percentile in measles incidence (Figure S5) and costs (Figure S6) to assess the potential hazard of overwhelming health and economic pressure stemming from a decrease in the MMR level within a distinct county. The results indicated that a mere 1% reduction in MMR vaccination rate within Richmond City could lead to a marked increase in measles risk in adjacent counties. The financial impact was notable, with deviations from the mean predicted costs surging to $100,000, accompanied by an increase in predicted measles incidence by 0.05 cases per 1,000,000 population. In contrast, an equivalent 1% decrease in MMR vaccination rate within Highland County manifested in a comparatively slight change in projected health and economic stress, with some counties experiencing less than 0.0001 additional incidence cases per 1,000,000 population from the mean. Meanwhile, in more distant counties, the variability in projected costs was minimal, deviating by only about $0.01 from the average anticipated costs. These results indicate the spatial variability of the impact of public health policies, showing epidemiological and financial volatility that recedes with distance from the origin of change in vaccination rate.

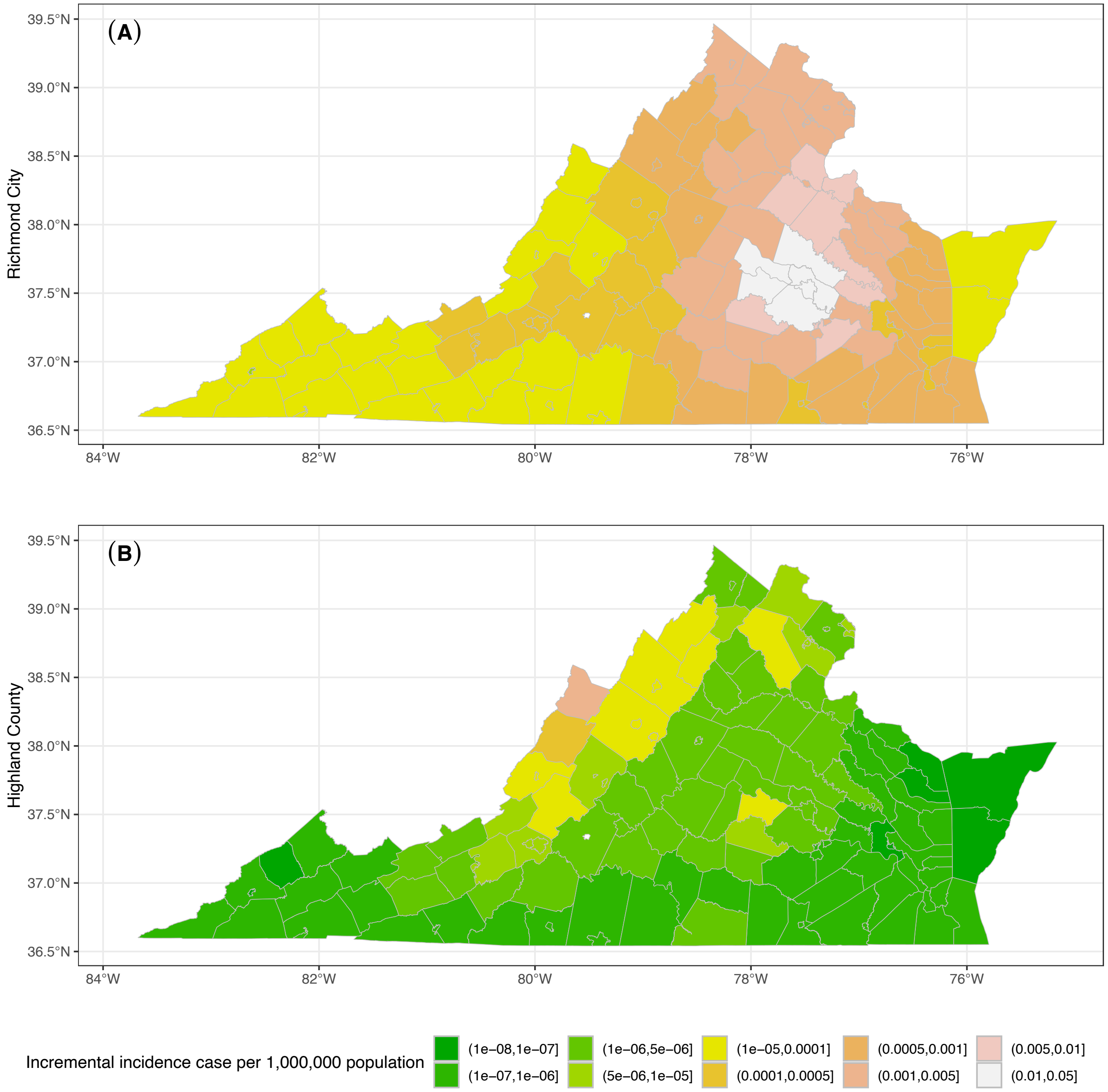

Figure S5. (A)Predicted 90th percentile incremental incidence case per 1,000,000 population when MMR level reduced by 1% (A) at Richmond City, or (B) at Highland County as a benchmark scenario.

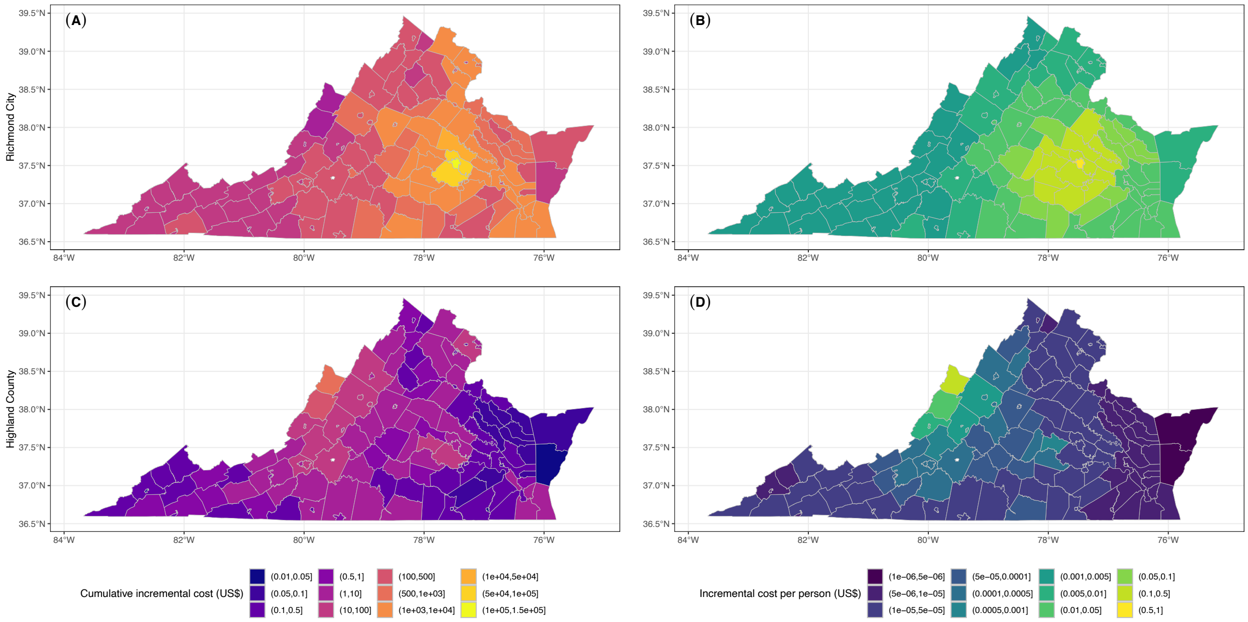

Figure S6. (Left) Predicted 90th percentile cumulative incremental cost and (Right) incremental cost per person (US$) when MMR level reduced by 1% at (Top) Richmond City and (Bottom) Highland County as a benchmark scenario, respectively.

5. References

[1] Pace JL Robert Kelley. Introduction to Spatial Econometrics. New York: Chapman and Hall/CRC; 2009. https://doi.org/10.1201/9781420064254.
[2] Gomez-Rubio V. Bayesian inference with INLA. CRC Press; 2020.
[3] Baltagi B, Song SH, Koh W. Testing panel data regression models with spatial error correlation. Journal of Econometrics 2003;117:123–50.
[4] Breusch TS, Pagan AR. The Lagrange Multiplier Test and its Applications to Model Specification in Econometrics. The Review of Economic Studies 1980;47:239–53. https://doi.org/10.2307/2297111.
